# GRASShopPER—An algorithm for *de novo* assembly based on GPU alignments

**DOI:** 10.1371/journal.pone.0202355

**Published:** 2018-08-16

**Authors:** Aleksandra Swiercz, Wojciech Frohmberg, Michal Kierzynka, Pawel Wojciechowski, Piotr Zurkowski, Jan Badura, Artur Laskowski, Marta Kasprzak, Jacek Blazewicz

**Affiliations:** 1 Institute of Computing Science, Poznań University of Technology, Poznań, Poland; 2 Institute of Bioorganic Chemistry, Polish Academy of Sciences, Poznań, Poland; 3 European Centre for Bioinformatics and Genomics, Poznań, Poland; 4 Poznań Supercomputing and Networking Center, Poznań, Poland; University of Helsinki, FINLAND

## Abstract

Next generation sequencers produce billions of short DNA sequences in a massively parallel manner, which causes a great computational challenge in accurately reconstructing a genome sequence *de novo* using these short sequences. Here, we propose the GRASShopPER assembler, which follows an approach of overlap-layout-consensus. It uses an efficient GPU implementation for the sequence alignment during the graph construction stage and a greedy hyper-heuristic algorithm at the fork detection stage. A two-part fork detection method allows us to identify repeated fragments of a genome and to reconstruct them without misassemblies. The assemblies of data sets of bacteria *Candidatus* Microthrix, nematode *Caenorhabditis elegans*, and human chromosome 14 were evaluated with the golden standard tool QUAST. In comparison with other assemblers, GRASShopPER provided contigs that covered the largest part of the genomes and, at the same time, kept good values of other metrics, e.g., NG50 and misassembly rate.

## Introduction

In the last decade, we have witnessed a dynamic evolution of a next generation sequencing (NGS). Its availability as well as potential has amplified, making it possible to reveal information from yet unknown genomes (e.g., panda [1], turkey [2], fungi [3], and bacteria [4]). Sequencers are now capable of producing billions of short DNA or RNA sequences, called reads, in a massively parallel manner. Consequently, sequencing takes no longer than a few days and costs far less than its precursor—the Sanger technology. However, like before, NGS reads come from random positions of a target genome sequence, and it is still a computational challenge to reconstruct the target sequence using only the information from the reads or pairs of reads in the case of the paired-end/mate-pair sequencing protocol. The process of such a reconstruction is called *de novo* assembly. Each *de novo* assembly method is based on a simple assumption that the reads cover the examined part of a genome and are overlapping one another. By utilizing the information from the overlaps, the method is able to reconstruct the genome sequence, approximately. However, if the reads do not cover the whole target, which is very likely because of technology limitations, the reconstruction may result in several independent sequences called contigs. Yet another problem the assembly method faces is repetitions of genome fragments. This may lead to misassembled genome fragments. From the computational point of view, the assembly can be seen as a more complicated version of the Shortest Common Superstring Problem, which is known to be NP-complete [5]. To make matters worse, the assembly problem has to tackle additional problems like sequencing errors, hardness in assigning reads to the proper DNA strand, or filtering the input data; for example, some reads may come from different organisms due to contamination.

Historically, one of the first methods to reconstruct a DNA sequence from shorter fragments was proposed in [6]. The method is related to sequencing by hybridization. This approach did not stand the test of time, but it initiated the industry of microarrays. The goal of the method was pretty much the same as in assembly nowadays, namely reconstruction of the original sequence from a set of its shorter fragments, here called *l*-mers (*l* stands for the length of the fragment). The only difference between *l*-mers and NGS reads is that the length of the former is much smaller. Lysov and coauthors modeled the problem as the graph theory problem of finding a Hamiltonian path. In the graph model, *l*-mers are associated with vertices and directed arcs represent their overlapping relations. Another solution of the problem was provided by Pevzner in [7]. He redefined the graph model to apply the Eulerian path problem, where *l*-mers are associated with arcs. Comparison of these two models was addressed ten years later in [8]. There the properties of labeled graphs, which are directed graphs with labels in vertices fulfilling special rules of overlapping, were analyzed, with de Bruijn graphs being one of its natural examples. Lysov graphs and Pevzner graphs are different kinds of subgraphs of de Bruijn graphs. More relationships between these graphs were explained in [9, 10].

The Pevzner graph model has been exploited in many algorithms for *de novo* assembly, which decompose reads into series of *l*-mers and benefit from such a reduction to the sequencing by hybridization problem (see e.g., [11–14]). In the literature, such a strategy is called, not quite justifiably, the de Bruijn graph approach (DBG). Nevertheless, in real world applications and in contrast to the original Pevzner’s solution, the occurrence of sequencing errors is inevitable, and the reconstruction problem becomes computationally harder. Only time- and memory-efficient heuristic algorithms are capable of dealing with large input data sets within a reasonable time. The overlap-layout-consensus strategy (OLC), based on the Lysov graph model, deals with the sequencing errors by allowing overlaps between pairs of reads represented by arcs to be inexact [15]. This approach has an advantage over the DBG strategy in the context of quality, as the latter approach partly loses the information about read continuity after the decomposition to *l*-mers. In DBG, paths corresponding to reads interweave and a heuristic algorithm is not able to strictly maintain their initial form. However, the quality improvement of OLC comes at the cost of memory consumption. OLC graphs (overlap graphs) need to store the information about overlaps, which is not necessary in DBG where *l*-mers themselves imply subsequent *l*-mers. In some circumstances, the quality of assembly results is the most crucial factor. For those cases, we find the OLC strategy promising. In fact, only a few assemblers utilizing this strategy have been developed, probably due to its computational cost [16–18].

On the other hand, new hardware technologies like graphics processing units (GPUs) offer much more computational power than CPUs. To focus our solution on producing high quality results in a reasonable time, we decided to use the OLC approach and parallelize its most time-demanding steps on GPUs. These ideas are the background of our new algorithm for de novo assembly GRASShopPER—GPU overlap GRaph ASSembler using Paired End Reads. A high performance module for overlap graph creation using graphics cards and a novel method for forks detection in the graph are the main features that differentiate GRASShopPER from other assemblers. We compare our algorithm with other top methods for *de novo* assembly: SOAPdenovo2 [13], Velvet [12], Platanus [14], Celera [16], String Graph Assembler (SGA, [18]), and SPAdes [19]. The data set consists of real NGS data for different types of genomes, starting from a bacterial genome to a repetitive mammalian genome.

## Results

### Algorithm overview

**Fig 1:**
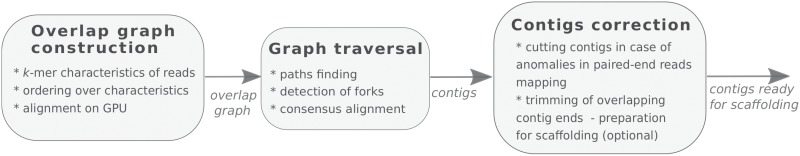
Diagram of the GRASShopPER assembler. The method has three main steps: construction of the overlap graph, its traversal, and correction of contigs.

GRASShopPER is composed of three main stages (Fig 1). At the beginning, an overlap graph is constructed from all high quality input reads including their reverse complementary counterparts (they constitute vertices of the graph). The part of calculating read overlaps is cut down to a reasonable minimum by a heuristic algorithm, which selects only those pairs of reads that are likely to overlap, the so-called promising pairs. The selection is based on the similarity of characteristics of corresponding *k*-mers (see section Methods for details). To confirm whether the promising pairs actually overlap, an exact algorithm for sequence alignment running on GPUs is executed. Later, in the overlap graph, arcs are created only for those pairs of reads having the number of misaligned residues on the overlapping section below an adjustable threshold. The graph is expanded by several improvements toward finding further promising pairs. For the constructed graph, the algorithm continues with a traversal method in order to find paths. The algorithm detects forks along its way, and unambiguous paths are translated into contigs. In the next step, the reads are mapped to contigs to find yet undetected forks by utilizing information from a wider context. This step allows reducing the misassembly rate and at the same time only slightly decreases the NG50 measure. At the end, the contigs are prepared for the scaffolding methods, which expect non-overlapping contigs.

### Other assemblers

We have compared GRASShopPER with a few assemblers that are well recognized in the scientific community: Velvet [12], SOAPdenovo2 [13], Platanus [14], SPAdes [19], Celera [16], and SGA [18]. They were used in the GAGE evaluation of assembly algorithms [20] and in the Assemblathon competitions [21, 22]. The first four assemblers represent the decomposition-based graph approach (DBG), and the rest are based on the idea of overlap graphs (OLC). Velvet, although developed in 2008 for very short reads, is still updated and works now with longer reads as well. It is one of the most popular assemblers and is still used in many *de novo* assembly projects [23, 24]. SOAPdenovo2 has been designed to be memory efficient and fast and still produces very high quality results. The origin of the tool lays in sequencing the giant panda genome [1]. Platanus was created recently to assemble highly heterozygous diploid genomes. While resolving bubbles in a de Bruijn graph, it distinguishes between repeated regions and heterozygosity and uses this information in the scaffolding step (a postassembly step for ordering non-overlapping contigs). The tool produced one of the highest NG50 values during the Assemblathon 2 contest and was used in several *de novo* assembly projects (e.g., [25]). SPAdes is the continuation of decomposition-based graphs proposed in [26] and paired de Bruijn graphs [27]. It uses multisized de Bruijn graph with different lengths of *k*-mers. It was tested mainly for small size genomes, like bacterial ones.

Methods following the OLC approach seem to be slightly underestimated. Nonetheless, the model is still developed in laboratories, which put high impact on the quality of the assembly results, where longer computation times or higher hardware requirements can be accepted. An example of this approach is the Celera assembler first released in 1999 [16] and then optimized under different names for different types of sequencers: wgs-assembler (Sanger/Applied Biosystems), CABOG (454/Roche), or PBcR (PacBio/Oxford). Another OLC-driven assembler under consideration is String Graph Assembler [18]. Here, read overlaps are calculated with the use of a compressed substring index based on the Burrows-Wheeler transform (FM-index), which can be effectively searched for the number of locations of a pattern within the compressed text. Additionally, the method transforms the graph into a so-called string graph by removing transitive edges. The method itself is memory- and time-efficient and was used in a few sequencing projects [28, 29]. We performed multiple tests for each algorithm in order to optimize the input parameter values for a reliable comparison of the methods.

### Data sets for tests

To achieve an unbiased comparison, we performed tests on three data sets differing in read length, coverage depth, and genome repetitiveness. The first data set is the actinobacteria *Candidatus* Microthrix parvicella, which can be commonly found in biological wastewater treatment plants. The draft of the genome consists of 4.2 Mb and is arranged in 13 scaffolds [23]. Raw reads were downloaded from the Sequence Read Archive (http://www.ncbi.nlm.nih.gov/sra, SRA058866, library accession number is SRX189748). The second data set comes from the nematode *Caenorhabditis elegans* strain N2 and was downloaded from the DNA Data Bank of Japan (http://trace.ddbj.nig.ac.jp/, accession number DRA000967). *C. elegans* provides a good test case for assembly methods, because of the completeness of its reference genome and reasonable size of 100 Mb. The third data set is one of the libraries provided in the GAGE benchmark, human chromosome 14 [20]. The data is available at GAGE web page (http://gage.cbcb.umd.edu/data/Hg_chr14/Data.original/frag_1.fastq.gz and http://gage.cbcb.umd.edu/data/Hg_chr14/Data.original/frag_2.fastq.gz). The length of chromosome 14 is estimated to be 107 Mb; however, due to a large gap of unknown nucleotides (N) at the beginning of the sequence, its effective length is approximately 90 Mb.

All the data sets contain paired-end read libraries of small insert size. The data were preprocessed before the assembly process. Illumina specific adapters were clipped. Reads containing ‘N’ or not mapping to the reference genome were removed from the libraries. Moreover, the reads were trimmed and filtered out, leaving those of the minimum average quality value of 30 along 60 consecutive nucleotides for *C. elegans*. Those values for *H. sapiens* were 20 along 30 nucleotides, and 30 along 60 for *C*. Microthrix. The summary of the read libraries and the size of the reference genomes are presented in Table 1.

**Table 1:** Characteristics of paired-end data sets of *C*. Microthrix parvicella strain Bio17-1, *C. elegans* and *Homo sapiens* after the preprocessing of raw reads with the adapter and low-quality trimming. * corresponds to the depth of coverage calculated for the length of the chromosome without a large gap of ‘N’.

genome	*C*. Microthrix 13 scaffolds	*C*. *elegans* 7 chromosomes	*H*. *sapiens* chromosome 14
species	bacteria	nematode	mammal
sequence length	4,202,850 bp	100,267,633 bp	107,043,718 bp
sequencer	Illumina GA II	Illumina GA IIx	Illumina HiSeq 2000
avg. read length	97 bp	109 bp	100 bp
no. of read pairs	2,463,704	30,436,661	12,015,343
avg. depth of cov.	113	66	26*
avg. insert size	312 bp	232 bp	159 bp
st. dev. of insert size	36 bp	56 bp	18 bp

### Evaluation metrics

Assembled sequences cannot be reliably evaluated with a single measure. There are a few complementary measures that need to be taken into account in the process of assembly method assessment. In the comparison, we chose the golden standard tool QUAST [30], which comes with a set of well-established metrics for the assembly problem. Genome fraction is one of the most important metrics here. It reveals the information on how much of the genome is covered by the provided contigs. The closer this value is to 100%, the greater number of reference nucleotides can be reconstructed from the contigs. The next relevant metric is the duplication ratio. It can be perceived, to some extent, as an orthogonal quantity to genome fraction. Duplication ratio helps to illustrate the redundancy of the information in contigs. Unfortunately, it does not take into account the repetitiveness of the genome itself, which may lead to an unjustified penalization of longer contigs just because they may cover alternative paths. One can view the assembly from yet another perspective by comparing the length of the largest alignment. Long continuous sequences are valuable in biological analysis. A similar rationale stands behind the NG50 and NGA50 metrics; however, they provide more comprehensive information not only from a single contig but also by being an aggregation from a number of longest ones. NG50 determines the length *c* of the longest contig that together with all the other contigs longer than *c* constitute, cumulatively, at least 50% of the genome length. The same applies to NGA50. This one, however, takes into account only these contigs that are successfully mapped to the reference genome. All the above metrics reward the maximization of the contig lengths. However, if we only try to extend contigs too heavily, we may end up with sequences too distinct from the target genome. One of the most important metrics exposing such errors is the misassembled contig length. It counts the length of all reported contigs that cannot be mapped continuously to the reference genome. In the evaluation of the assembly results we set the maximum misassembled contig length as 1 percent of the reference genome length. The methods having greater misassembled length were not considered in the scaffolding phase. It is noteworthy that QUAST filters out too short contigs, considering them as relatively uninformative, in our tests, we set the contig limit to 250 bases. The detailed summary of all the used metrics can be found at QUAST manual page (http://quast.bioinf.spbau.ru/manual.html). The heatmap presented in Table 2 is derived directly from QUAST.

**Table 2:** Assemblies obtained for three data sets: *C*. Microthrix, *C. elegans*, and human chromosome 14 (metrics calculated by QUAST).

Genome statistics	GRASShopPER	Celera	Platanus	SGA	SOAPdenovo2	Velvet	SPAdes
Data set of Candidatus Microthrix parvicella strain Bio17-1							
Genome fraction (%)	98.73	89.21	98.38	98.82	98.52	97.86	98.96
Duplication ratio	1.006	1.002	1.017	1.002	1.001	1.000	1.001
Largest alignment	126,696	50,960	25,116	101,782	107,154	166,835	740,450
Total aligned length	4,173,839	3,755,338	4,203,934	4,161,932	4,145,584	4,113,131	4,161,980
NG50	33,570	11,255	5,286	32,697	34,653	78,563	156,137
NG75	16,714	5,614	2,889	18,691	17,879	39,191	104,295
NGA50	33,566	11,013	5,281	32,697	34,653	77,856	151,220
NGA75	16,712	5,528	2,886	18,691	17,879	39,191	88,932
# misassembled contigs (length)	4 (11 kb)	4 (32 kb)	1 (6 kb)	3 (37 kb)	1 (31 kb)	6 (330 kb)	5 (370 kb)
no. contigs (> 0 bp)	439	493	2,107	668	949	103	1,297
no. contigs (≥250 bp)	336	449	1,395	257	267	103	808
no. contigs (≥ 1 kb)	254	424	966	215	220	103	64
no. contigs (≥ 5 kb)	159	256	257	161	157	88	49
no. contigs (≥ 10 kb)	112	127	66	118	115	75	44
no. contigs (≥ 25 kb)	57	21	1	56	54	51	30
no. contigs (≥ 50 kb)	19	1	0	20	21	29	23
Data set of Caenorhabditis elegans							
Genome fraction (%)	95.47	78.81	88.39	93.92	92.58	85.61	94.81
Duplication ratio	1.019	1.020	1.004	1.008	1.004	1.004	1.004
Largest alignment	96,261	33,627	63,884	80,404	83,885	58,073	180,696
Total aligned length	97,504,793	80,514,782	88,972,062	94,936,888	93,192,365	85,981,341	95,338,850
NG50	7,772	3,982	4,157	6,618	6,364	7,000	20,063
NG75	2,793	1,789	1,402	2,665	2,486	3,018	8,732
NGA50	7,771	3,903	4,088	6,581	6,313	6,736	18,679
NGA75	2,783	1,700	1,277	2,557	2,325	2,576	7,495
# misassembled contigs (length)	142 (177 kb)	524 (2402 kb)	5 (47 kb)	55 (215 kb)	12 (58 kb)	337 (2229 kb)	475 (8519 kb)
no. contigs (> 0 bp)	82,283	21,503	233,557	150,360	160,015	17,510	52,752
no. contigs (≥ 250 bp)	38,336	20,766	42,224	34,185	33,847	17,510	13,779
no. contigs (≥ 1 kb)	15,971	20,220	20,742	18,911	19,006	17,510	9,320
no. contigs (≥ 5 kb)	5,108	4,912	4,328	5,246	5,100	5,897	4,915
no. contigs (≥ 10 kb)	2,247	1,108	1,572	2,122	2,004	2,278	2,866
no. contigs (≥ 25 kb)	401	17	167	287	307	202	946
no. contigs (≥ 50 kb)	39	0	6	14	17	5	244
Data set of human chromosome 14							
Genome fraction (%)	92.28	75.96	71.80	88.30	88.99	72.33	93.53
Duplication ratio	1.038	1.005	1.005	1.007	1.007	1.007	1.011
Largest alignment	38,022	39,634	13,122	30,294	28,332	41,564	58,597
Total aligned length	86,648,380	69,025,599	65,344,557	80,527,423	81,146,875	65,606,013	85,602,007
NG50	2,500	2,891	782	2,909	2,418	2,628	4,755
NG75	1,020	1,154	-	1,207	1,000	-	2,260
NGA50	2,500	2,891	782	2,909	2,418	2,628	4,755
NGA75	1,014	1,077	-	1,202	997	-	2,204
# misassembled contigs (length)	123 (207 kb)	1110 (5039 kb)	0 (0 kb)	51 (171 kb)	17 (92 kb)	358 (1606 kb)	559 (3601 kb)
no. contigs (> 0 bp)	81,314	21,003	574,441	97,520	239,297	21,153	62,461
no. contigs (≥ 250 bp)	64,638	20,930	72,766	40,353	48,947	21,153	29,935
no. contigs (≥ 1 kb)	24,310	20,880	21,035	22,930	24,237	21,153	19,521
no. contigs (≥ 5 kb)	2,988	3,607	259	3,460	2,830	3,398	4,796
no. contigs (≥ 10 kb)	468	559	1	525	390	651	1,354
no. contigs (≥ 25 kb)	7	4	0	12	1	10	105
no. contigs (≥ 50 kb)	0	0	0	0	0	0	1

### Assembly of the Microthrix bacteria

Table 2 presents the comparison of assemblies obtained for the three data sets. In the first part of the table, the results on the Microthrix bacteria data set are shown. The first observation is that Celera results seem to be inferior with reference to the three collectively relevant metrics: genome fraction, largest alignment, and NG50. On the other hand, it generates the largest number of contigs that are longer than 10 kb. One could also notice that SPAdes produces the longest contigs, which is reflected in both largest alignment and NGA50. However, this payoff appears to be at the cost of quality—SPAdes has the highest number of misassembled contigs, having significantly larger cumulative length (e.g. 10 times larger than SGA has). Velvet obtained the second longest aligned contigs and NGA50 value. Similarily to SPAdes, contig length bloat induced radical increase of observed misassembly rate in comparison to other methods. Another conclusion can be drawn from Platanus results. Basing on relatively low NG50 and extremely short largest alignment, one can get the impression that its contigs are highly shredded. Still, the results of Platanus are interesting, because of a decent genome fraction and high quality results with a very low misassembly rate. SGA, SOAPdenovo2, and GRASShopPER produce results with quite similar reasonable values on all considered metrics. They all cover nearly 99% of the reference genome. The largest alignment of these methods exceeds 100 kb.

**Fig 2:**
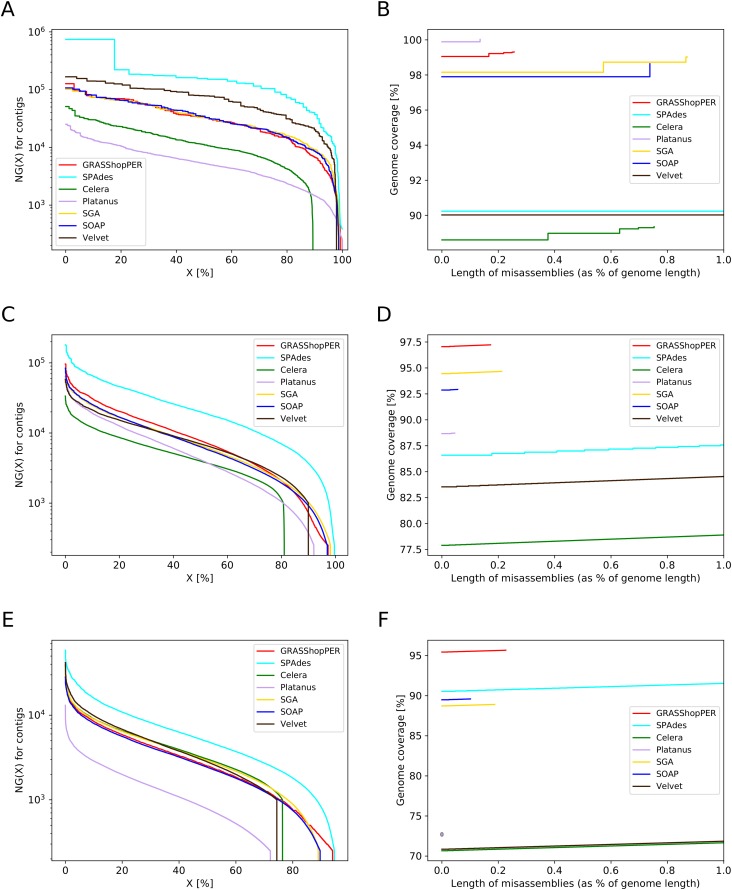
Values of NG(X) and the genome coverage as a function of the misassembled contigs length. (A) Values of NG(X) for *C*. Microthrix data set. (B) Genome coverage and misassemblies for *C*. Microthrix data set. (C) Values of NG(X) for *C. elegans* data set. (D) Genome coverage and misassemblies for *C. elegans* data set. (E) Values of NG(X) for human data set. (F) Genome coverage and misassemblies for human data set.

Some of these remarks can also be observed in Fig 2A and 2B. The first figure exposes correlation between the distribution of the NG(X) lengths and the genome coverage, while the second one is a function of the genome coverage and the length of misassembled contigs for each assembler. The NG(X) is a length of the contig that combined with all longer contigs covers X% of the genome. For example, Platanus covers 20% of the genome with contigs of length 10 kb or longer, while for SGA, SOAPdenovo2, and GRASShopPER the contig length is almost 100 kb. Each line drops down to zero on the right side of the graph in the place corresponding to the aggregated length of all contigs. The rugged lines in this graph are due to the fact that the longest contigs cover a significant percentage of this short genome, which is expressed as the steps, more visible on the left side of the graph where larger contigs are placed. The figure is informative, but one should not evaluate the methods based only on it. Otherwise, one would give the highest score to SPAdes and the lowest to Platanus. What we observed previously in Table 2, by looking at the misassembly rate, was the opposite. Also the second figure (Fig 2B) confirms that misassembled contigs highly impact on the genome coverage. The X axis stands for the misassembled contig lengths that are taken into account in calculating the genome coverage. Zero means there no tolerance for misassemblies—this corresponds to the rigorous situation when each partly valuable information concealed in a contig should not participate in the genome coverage. On the right-hand side of the chart we restricted the misassembled contig length to 1 percent of the genome length, assuming that greater values lead to too low quality results. The observable steps in the chart originate from the length of misassembled contigs, the longer the contig, the longer the step is. On the other hand, the height of the step depends on the length of the reference genome fragment it covers. For example, the longest misassembled contig produced by SGA is almost of 0.6% of genome length, that is why the first step starts around 0.6. Platanus has very low misassembly rate, and its line ends at approximately 0.15% of the genome length. SPAdes and Velvet output very high misassembly rate, approximately 8% of the genome length, and the longest misassembled contig exceeds 1%. This is the reason why we observe just the straight lines for the two methods without any steps. Only three methods: GRASShopPER, SGA and SOAPdenovo2, which are in the middle of the NG ranking, yet not superior for any measure, provide a balanced trade-off between contigs/NG lengths and the assembly quality/genome fraction.

### Assembly of the nematode *C. elegans*

The genome of Microthrix is relatively small. In this context, the *C. elegans* data set represents a more challenging test case (results are presented in the second part of Table 2). It is quite obvious that a longer genome entails a longer overall length of misassemblies; however, results of SPAdes, Celera and Velvet in this matter are a bit disturbing. SPAdes obtained misassembled contigs of length over 8.5 Mb, while both, Celera and Velvet, produced bad contigs of cumulative length exceeding 2 Mb, which for the genome consisting of approximately 100 Mb is considerably below expectations. Even bigger disappointment comes from the Celera contigs genome fraction, which together with its outlying result on the largest alignment and NG50 makes Celera inferior on this data set. The same does not apply to Velvet, which, although also has relatively low genome fraction, provides a satisfactory NG50 value. On contrary, SPAdes outputs the largest alignment, the highest NG50 value, and second best genome fraction. Platanus acts here a bit unexpectedly, as it does not cover the genome to a large degree (only 88.4%). On the other hand, it has remarkably low misassembled contig length. Again, it is hard to find the favorite between SGA and SOAPdenovo2. They both cover the genome well and produce quite long contigs, while SOAPdenovo2 has a bit better quality. This time GRASShopPER beats all its opponents in the genome fraction, keeping high values of largest alignment, NG* statistics, and the number of contigs longer than 50 kb. However, the method results in a little higher rate of duplicated information inside contigs. The distribution of NG lengths presented in Fig 2C confirms that, although, SPAdes outperforms other assemblers in NG(X) metrics due to forcing to contig length bloat, it suffers from poor misassemblies metrics (Fig 2D) and thus provides results of a lower quality. Apart from SPAdes, also Velvet and Celera give a high misassembly rate, which drastically decreases genome coverage when considering only good quality contigs.

### Assembly of human chromosome 14

The last tests were performed to provide reliable quality assessment of all the methods on an even more challenging data set, the 14th human chromosome. The tests confirmed that Platanus scatters its contigs to a large degree, at the same time yielding an incredible quality result without a single misassembled contig. The low genome fraction rate might be due to the fact that only contigs longer than 250 bp are taken into account by QUAST. In the case of Platanus, only 13% of produced contigs (574 k) are longer than the minimum meaningful length. The second method that produced a high number of very short contigs was SOAPdenovo2, for which only 20% of contigs were longer than 250 bp.

The data set of human chromosome 14 has the lowest depth of coverage among the three tested sets. Most likely, this is the reason for the smallest genome fraction and NG values, although the lengths of human chromosome 14 and of the *C. elegans* genome are similar. Nevertheless, SPAdes and GRASShopPER have become a leader, covering 93.5% and 92.3% of the genome, respecitively, 4-5% more than the SGA and SOAPdenovo2 assemblers, and approximately 20% more than the other three methods. This can be easily observed in Fig 2E, by the sudden drop down to zero for NG lengths, much before the GRASShopPER method. Among the assemblers covering more than 80% of the chromosome, SPAdes and SGA are outstanding in NG values, although SPAdes achieved this high rank at the cost of a very high misassembled contig rate (3.4% of the chromosome length). NG75 values could not be computed for Platanus and Velvet, as their genome coverage is below 75%. We can observe that the longest alignments, observed for SPAdes, Velvet and Celera, strongly affect the length of misassembled contigs (Table 2 and Fig 2F). In this context, GRASShopPER is the best assembler that does not exceed with its misassembled contigs 1% of the chromosome length.

The three data sets provide a wide variety of benchmark test cases. The libraries vary in the reference genome length and coverage. We could see that the methods which are superior in one or two metrics are the worst in the others. Some of the methods were working well for one of the data sets (e.g., Velvet for the high coverage data set of the Microthrix bacteria) but resulted in much lower genome fraction for other sets. Moreover, none of the methods could be seen as the worst in terms of one criterion in all tested cases. All of the metrics provided by QUAST are presented in S1–S3 Tables.

We selected two assemblers that achieved high genome coverage and behaved reasonably well on other metrics (where misassembled contig length did not exceed 1% of the genome length) across the tested instances, SGA and SOAPdenovo2. These two methods were further compared with GRASShopPER in the scaffolding phase.

### Scaffolding

In the last phase of the process of reconstructing a genome, scaffolding, contigs may be further joined on the basis of paired-end reads. We used freely accessible scaffolding tools [31], and chose two of them that produce the best results for the selected assemblers: SSPACE [32] and SOAPdenovo2 scaffolding module. SSPACE is a greedy approach. It starts from extending the longest contig as long as there are some read pairs supporting the extension. Next, it continues with the remaining largest contig. SOAPdenovo2 is a topology-based approach that establishes a relationship between the contigs. Heterozygous contigs are detected and only the contigs with higher depth of coverage remain in the scaffolds, which reduce the impact of heterozygosity on the scaffold lengths.

In Table 3, we provide the results for the best combinations of assembler and scaffolder. The remaining results are accessible in S4–S6 Tables. While choosing the best scaffolder for each assembler, we were not taking into account just a single metric. We wanted the scaffolds to be long and of high quality, i.e., not providing a large amount of misassemblies. Hence, the selected scaffolder is not always the same for a given assembler for the tested data sets.

**Table 3:** Scaffolding of the three data sets for the assemblers GRASShopPER, SOAPdenovo2, and SGA with the best combination of scaffolders SSPACE and SOAPdenovo2 (metrics calculated by QUAST).

Assembler	Scaffolder	Genome fraction (%)	Largest alignment	Total aligned length	NG50	NG75	Misassembled scaffolds (length)
Data set of *Candidatus* Microthrix parvicella strain Bio17-1							
GRASShopPER	SSPACE	98.602	**126,696**	**4,165,836**	33,570	16,714	**4 (11 kb)**
SGA	SSPACE	**98.819**	101,782	4,161,932	32,697	**18,691**	3 (37 kb)
SOAPdenovo2	SOAPdenovo2	98.522	107,154	4,145,584	**34,653**	17,879	1 (31 kb)
Data set of *Caenorhabditis elegans*							
GRASShopPER	SOAPdenovo2	**95.357**	**126,121**	**97,363,265**	11,352	4306	461 (1,2 Mb)
SGA	SOAPdenovo2	94.037	105,248	94,990,734	10,187	4149	443 (1,2 Mb)
SOAPdenovo2	SSPACE	93.549	119,149	93,929,080	**12,859**	**4920**	**95 (838 kb)**
Data set of human chromosome 14							
GRASShopPER	SSPACE	**92.275**	**38,022**	**86,645,202**	2500	1020	123 (207 kb)
SGA	SSPACE	88.586	35,224	81,213,834	**3040**	**1289**	114 (268 kb)
SOAPdenovo2	SOAPdenovo2	89.582	33,186	81,612,904	2894	1206	**31 (130 kb)**

In the tests of the scaffolding phase, we were using contigs produced by GRASShopPER with the postprocessing step switched on, which reduced the redundancy at the ends of contigs. The necessity of this additional step is required, because scaffolders do not take into account that the ends of contigs might overlap and align them one after another. This produces an extra few dozen of nucleotides repeated twice inside a scaffold. This issue is explained in detail in the Methods section.

**Fig 3:**
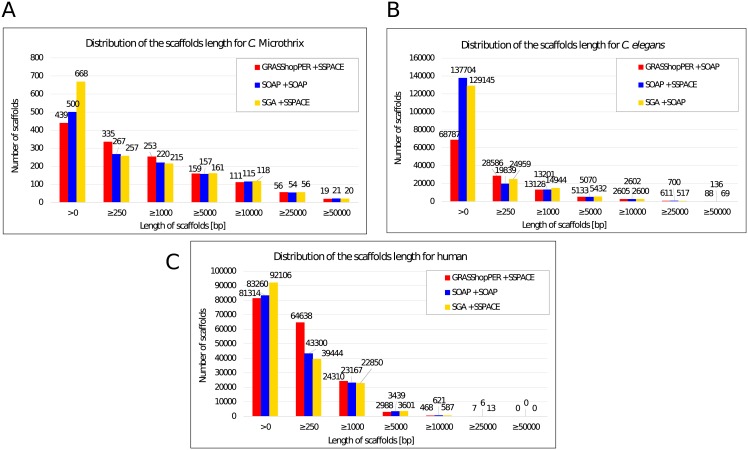
Numbers of scaffolds. (A) Number of scaffolds of a given length for *C*. Microthrix data set. (B) Number of scaffolds of a given length for *C. elegans* data set. (C) Number of scaffolds of a given length for human data set.

A few selected metrics for the scaffolding results are presented in Table 3, while the distribution of the number of scaffolds of a given length is presented in Fig 3A, 3B and 3C.

In the first part of Table 3, the summary results of scaffolding for the *C*. Microthrix data set are given. Both scaffolders, SOAPdenovo2 and SSPACE, gave similar results (see S4 Table) for all assembly methods, which, additionally, were not very different from the original contigs. Genome coverage, NG statistics, and the largest alignment remained the same as for the input contigs (in the case of GRASShopPER, compare the contigs after the postprocessing step in S4 Table). The only difference was in reducing the number of very small contigs (< 250 bp) in the case of the combination of the SOAPdenovo2 assembler and a scaffolder. These contigs were either merged into scaffold sequences, which could not be observed by the generalized statistics, or rejected by the scaffolder. The reason for the invariability of the results before and after the scaffolding phase might be the rather short genome length, 4 Mbp, and its division into 13 fragments, its low repetitiveness, and high coverage of the input reads. The assemblers have obtained contigs that were not further elongated by the scaffolders.

The second part of Table 3 and S5 Table provide the results of scaffolding for the *C. elegans* data set. In this case, the scaffolders have improved the results for every input contig set. The best combination of greatest NG values and smallest misassembled scaffold length was obtained for the SOAPdenovo2 assembler with the SSPACE scaffolder, although, the misassembly rate is much higher than for the assembly results (838 kb vs. 58 kb). Both GRASShopPER and SGA worked better with the SOAPdenovo2 scaffolder, obtaining NG50 lengths of 11 kb for GRASShopPER and 10 kb for SGA. The cumulative length of misassembled scaffold sequences reached the value 1.2 Mb for both methods. The postprocessing step for GRASShopPER, which cut the overlapping ends of the contigs, allowed reducing significantly the misassembled length from 3.6 Mb (see S5 Table). GRASShopPER again outperformed all the methods in the genome coverage and the aligned scaffold lengths.

The last part of Table 3 and S6 Table provide the comparison of scaffolding made for human chromosome 14. Scaffolders were not able to improve results of GRASShopPER. However, its highest rate of genome coverage and largest alignment is preserved. The greatest NG50 was achieved this time by the combination of the SGA assembler and the SSPACE scaffolder. It should be noticed that even though the lengths of the *C. elegans* genome and human chromosome 14 are similar, their statistics are very different. The NG50 scaffold length for *C. elegans* is approximately 11 kb, while for human it is hardly more than 3 kb in the best case. The human genome is more repetitive, and, in this specific data set, we had a much smaller depth of coverage than for *C. elegans*. Thus, there might be many more places with much lower read coverage, which prevents us from reconstructing longer contiguous sequences.

### Test environment

The computational tests were done in the Poznan Supercomputing and Networking Center on a cluster named moss, which is a part of the Polish Grid Infrastructure (PL-Grid). Moss provides six highly specialized nodes for heterogeneous computing, each equipped with two general purpose graphics processing units (GPGPUs), between 256 and 512 GB RAM, and two CPUs. Whenever possible, all the methods were run on one node with 16 cores. The parameters of all the methods used in the computational experiment are given in S1 Text.

Resources used in the assembly process of *C*. Microthrix, *C*.*elegans*, and *H*.*sapiens* chromosome 14 by GRASShopPER were 25 minutes/17 GB RAM in peak, 4579 minutes/335 GB RAM in peak, and 603 minutes/82 GB RAM in peak, respectively. For other assemblers, the time and memory requirements were (in peak, either for *C. elegans* or *H. sapiens* chr. 14): 2270 minutes/422 GB RAM for Celera, 519 minutes/65 GB RAM for SPAdes, 370 minutes/29 GB RAM for Velvet, 124 minutes/7 GB RAM for SGA, 76 minutes/15 GB RAM for Platanus, and 33 minutes/11 GB RAM for SOAPdenovo2.

## Discussion

We are reporting on GRASShopPER, an overlap graph assembler employing GPUs, which uses information from paired-end reads for resolving repetitions in a genome sequence. GRASShopPER is based on the OLC approach, which does not lose information by decomposing input reads into *k*-mers, but, at the same time, is more time and memory demanding than the DBG approach. We use a very efficient GPU implementation of the reads alignment algorithm for calculating the scores and shifts on the arcs of the graph. We introduce a two-part fork detection strategy, which highly reduces misassemblies in the resulting contigs. The first part is carried out during the traversal of the graph. In the second part, a greedy hyper-heuristic finds undetected forks on the basis of paired-end read information.

The assemblies of the data sets of bacteria *C*. Microthrix, nematode *C. elegans*, and human chromosome 14, were evaluated with the golden standard tool QUAST. We observed that GRASShopPER produced contigs that covered the largest part of the genomes (metric ‘genome fraction’) and usually had a few percent more coverage than other methods (Table 2). The largest difference was observed in the case of the data set of human chromosome 14 with the lowest depth of coverage, for which Platanus obtained even 20% less of the genome fraction coverage than our method. Among the tested assemblers, Velvet and Celera produced contigs with the lowest coverage and highest misassembly rate. On the other hand, both methods were superior on NG(X) length—Velvet for *C*. Microthrix and Celera for human chromosome 14 data set. SPAdes for all tested data sets output the longest alignments and the highest NG50 and NG75, but at the same time the quality of these contigs was very low—the assembler gives the greatest misassemblied contig lengths, reaching in total even 8.5% of the genome length. Platanus, and in some cases also SOAPdenovo2, produced a huge amount of contigs shorter than 250 bp, which was the minimum length threshold for QUAST, and thus were not considered in the statistics. This highly influenced the lower genome fraction of Platanus. However, one may wonder if the information given in 500 thousand contigs (in the case of the human data set), a little longer than the input reads, is of any value. The two methods, Velvet and Celera, output only (or mostly) contigs longer than 1 kb. This fact could be easily observed in Fig 2, which shows that at NG(X) equal to approximately 1 kb, there is a sudden drop of the line toward zero. The other three methods, GRASShopPER, SOAPdenovo2, and SGA, behave reasonably well for all considered statistics and for all tested data sets, providing high quality and long contigs that covered the largest part of the considered genomes.

The further step of scaffolding with the use of external tools SSPACE and SOAPdenovo2 revealed that only for the *C. elegans* data set the scaffolding tools were able to markedly improve the results by merging and lengthening the contigs. In the case of this data set, the SOAPdenovo2 assembler worked better with the SSPACE scaffolder, while GRASShopPER and SGA gave better results with the SOAPdenovo2 scaffolder. For the other data sets, with less depth of coverage, it was the opposite—GRASShopPER and SGA worked better with SSPACE scaffolder and SOAPdenovo2 assembler with its original scaffolder. Results obtained for all combinations of the tools are listed in S4–S6 Tables.

Among the three best performing assemblers, SGA, SOAPdenovo2, and GRASShopPER, the latter one achieved the highest rates in the context of genome coverage and alignment length for *C. elegans* and human chromosome 14, with a moderate misassembly rate. For *C*. Microthrix, it keeps its position regarding the largest alignment, with the lowest misassembly rate, and shares the leadership on other metrics.

Although the new method presented in the article is not the most efficient among the tested assemblers with respect to memory and time usage, it represents the overlap-layout-consensus strategy, which is considered to be more accurate. Due to its precision, the OLC strategy requires more time and memory resources. It is especially noticeable in the graph construction step, where the method needs to remember reads on vertices and calculate the alignment of the pairs of reads to decide to what extent the reads overlap one another. Moreover, the method does not lose the information about reads continuity caused by the decomposition into shorter *k*-mers, as entire reads are stored in the graph vertices. This is particularly important in the case of repetitions of short DNA fragments. We find the OLC strategy to be the future of the assembly of long read data sets produced by third generation sequencers, such as, for example, Pacific Biosiences or Oxford Nanopore.

## Methods

### Overlap graph construction

In the overlap graph, every read and its reverse complementary counterpart are represented by a double vertex. To differentiate a read with distinct paired-end reads, even if the read sequences are identical, they are considered separately. The goal of the graph construction is to connect vertices by arcs when corresponding reads overlap. The problem is not only in erroneous reads, which impose the need for inexact comparison, but also in their number. Because of the latter, it is not feasible in practice to compare every sequence with each other. Therefore, a method is needed for an efficient preselection of overlapping reads. The most important step of the preselection adopts the *k*-mer characteristic as an indicator of the similarity between reads. To be more precise, given the length *l* of a read, the maximum number of extracted *k*-mers is equal to *l* − *k* + 1. However, if a *k*-mer occurs multiple times within a sequence, it appears only once in its characteristic—but with an increased counter. After extracting *k*-mers from a sequence, they are sorted internally (within each characteristic) in descending order according to their numbers of occurrence. Next, all the *k*-mer characteristics are sorted alphabetically, just like words in a dictionary. As the most distinguishing *k*-mers were put in front, a chance that neighboring characteristics refer to overlapping sequences is very high. We observed that if two sequences overlap on at least half of their length, then their *k*-mer characteristics are very close to each other once sorted. On the other hand, the characteristics tend to drift apart for those sequences that overlap on a relatively short segment only. To address this issue, we introduced partial characteristics in which *k*-mers are extracted only from selected parts of a sequence, i.e, the beginning, center, or the end of a read. The sorting procedures and further steps are the same as in the first scenario.

Pairs of reads laying within a given neighborhood are called *promising pairs*. The size of the neighborhood on the sorted lists is a parameter of the method. To verify the overlapping property of the promising pairs, we align the corresponding pairs of reads using a semi-global version of the Needleman-Wunsch algorithm (NW), which finds optimal alignments. This is a relatively time-consuming method, but it is applicable since we use a very efficient implementation on the GPU platform, the G-DNA library [33]. At the output of the method, we get the alignment score and the so-called shift value for each pair of tested reads. Those pairs, for which the overlap is sufficiently long and the number of alignment errors is below a given threshold, are connected by an arc in the graph model.

**Fig 4:**
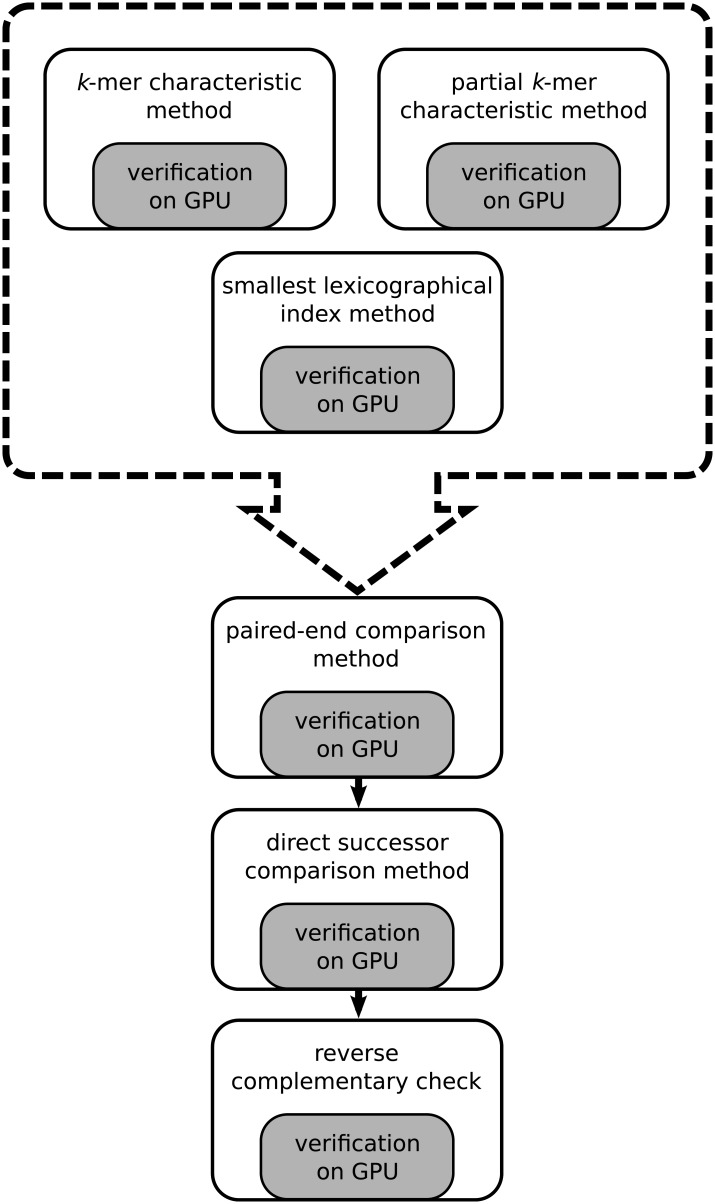
Graph construction algorithm.

As already mentioned, the *k*-mer characteristic-based selection is only one of the steps to preselect pairs of reads that are likely to overlap. A sketch of the entire method is presented in Fig 4. One of the next steps is the smallest lexicographical index method. It selects two descriptors for each read, one for the first half and the other for the second half of a read. The descriptor is defined here as the lexicographically smallest subsequence of a fixed length, usually between 12 and 20 nucleotides. Reads having the same descriptors are marked as promising pairs and their overlapping is verified with the G-DNA library. The next step takes into account the information given in paired-end reads. Assume we have two paired-end reads: *A* paired with *A*′ and *B* paired with *B*′. If *A* and *B* overlap, then *A*′ and *B*′ may overlap as well. Therefore, all such pairs (*A*′, *B*′) become promising and the algorithm verifies their alignment. The next step compares direct successors of vertices. Let *A*, *B* and *C* denote reads. If *A* overlaps *B* and *A* overlaps *C*, then *B* and *C* are marked as a promising pair and the GPU-based algorithm verifies the quality of the alignment. The last step verifies the reverse complementary sequence alignment. Let *A* and *B* denote sequences, and let $\bar{A}$ and $\bar{B}$ be their reverse complementary counterparts, respectively. If *A* and *B* do overlap, then $\bar{A}$ and $\bar{B}$ must overlap too. In this case, alignment-based verification is needed only to check a shift between reads to discover an exact number of errors inside the alignment (without the expensive procedure of backtracing).

### Graph traversal

When the overlap graph is created, the GRASShopPER algorithm can start the traversal phase in search of unambiguous paths. In case of graphs for complicated genomes (containing many repetitive regions) constructed on erroneous sequence sets, we may encounter a number of difficulties to detect ambiguities. Those fragments of the graph usually form fork-like structures, where at one point of the search phase one must choose between two or more paths that are equally possible. The forks being a result of sequencing errors must be ignored or carefully handled.

The traversal algorithm selects multiple random starting points for contigs and extends them in both directions. Instead of a single vertex, we operate on a set of vertices, called the *state*, which were recently added to the path. Vertices from the state vote for candidates for extension, being their direct successors, and a weighted sum of the votes determines the score of a candidate. Therefore, it is not likely that a single wrongly chosen vertex affects the whole path. Every iteration of the traversal algorithm ends with the selection of the candidate with the highest score and adds it to the state.

Let *S* = *s*_1_, *s*_2_, …, *s*_*r*_ be a state, which is a list of *r* most recently added vertices *s*_*i*_ to the currently traversed contig and let *C* be a list of candidates considered as successors. $C=\cup_{i=1}^{r}out\left(s_{i}\right)$, where *out*(*s*_*i*_) is the set of vertices being successors of *s*_*i*_ in the graph. The size *r* of the state is dynamically adjusted based on the graph density and the level of overlaps of vertices being considered as a premise to form an arc. Whenever the overlap between *s*_1_ and *s*_*r*_ is smaller than the required sequence overlap between vertices from the state, the oldest vertex in the state (*s*_1_) is removed. This way the state contains all and only the meaningful vertices—the ones that can contribute to the selection.

The candidate successor is chosen on the basis of the scoring function, which weights the votes of the predecessors of candidates by the level of similarities concealed in arcs. The scoring function sums the overlaps of all the supporting arcs and, therefore, promotes candidates supported by newest vertices in the state.

### Fork detection

The main source of forks in DNA overlap graphs is the occurence of errors in the sequenced reads, single nucleotide polymorphisms (SNPs), and repeated regions of a genome. All three cases result in similar graph structures, but it is possible to differentiate among them. Sequencing errors are the easiest to detect and resolve, because they most often have a very low confirmation in the neighboring sequences, e.g., one of the branches ends up shortly after the fork, while in case of repetitions it does not. SNP branches join back together right after the fork, so it is possible to detect them too. When it comes to repetitive fragments, one needs to be very cautious, because both paths may appear to be equally accurate. Unfortunately, looking from the whole genome perspective, further traversal might lead to incorrect contigs. Therefore, the algorithm must stop traversing the path each time an unsolvable fork is detected. Following a random path on consecutive forks most often results in a misassembly. In the rest of the section, we focus mainly on branches that come from repetitive fragments.

The algorithm detects two types of forks: forward and backward. A forward fork occurs when graph traversal along one path creates a possibility of going to two or more different paths. A backward fork occurs when two (or more) paths merge into one in the graph.

**Fig 5:**
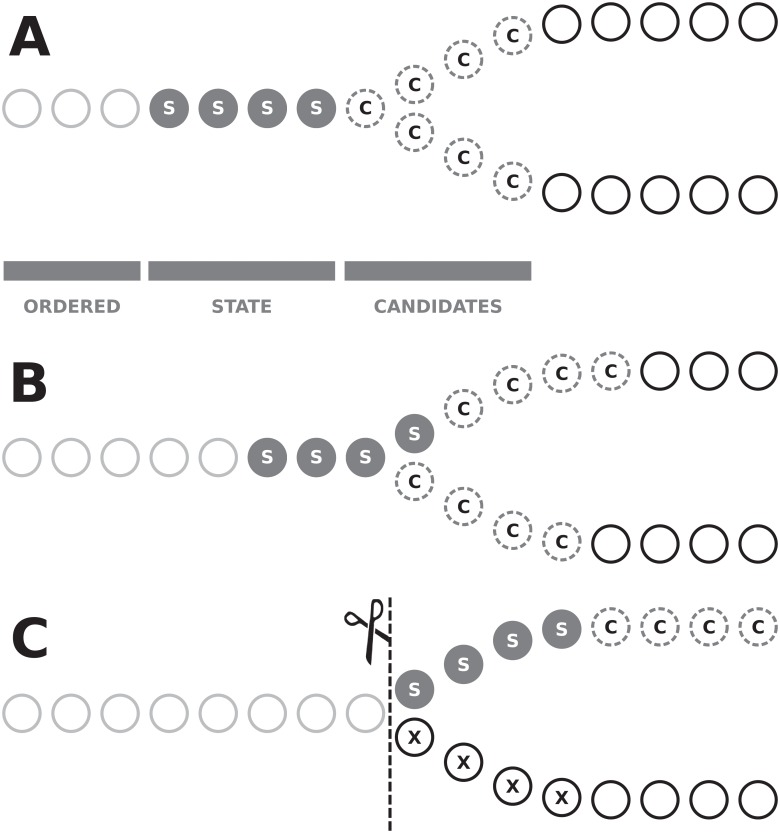
An example of the fork detection made by the algorithm. The ordered vertices are already in a path (A). Vertices from the state are in the path and they vote for the candidates, which could extend the current path (A, B). When all the vertices from the state are moved toward one branch of the fork, and many candidates from the other branch are lost, the algorithm cuts the current path at the beginning of the fork (C).

While approaching a forward fork, *C* contains vertices from two (or more) paths (Fig 5A). At this point, we are not aware of the existence of a fork ahead of us, the fork is detected once the whole state is placed entirely on one of the paths after crossing the branch and there is no candidate on the other paths. When the first candidate from one of the distinct paths is chosen (Fig 5B), the scores on all branches are modified and higher scores are assigned to candidates on the selected branch. Hence, they are more likely to be selected as a subsequent contig vertices. When the last vertex that has successors in more than one branch is removed from the state, a group of candidates that belong to the other path (or paths) is also removed (Fig 5C). This confirms the existence of a fork and suggests a cut of the path directly before the state. However, if not all arcs are detected during the graph construction phase and dropped vertices have connections to the vertices from *S*, we may mistakenly detect a fork while the dropped vertices should be included in the current path. Thus, it is necessary to countercheck that there is no arc between vertices from the removed group to the state, otherwise we may mistakenly detect a ‘jump’ over skipped vertices.

Detection of the backward fork follows similar rules as for the forward fork. Compared to forward forks, which expect the removal of candidates—backward forks expect the addition of candidates in the situation when the state enters the vertex common for both branches of the backward fork. To confirm the fork—for the same reason as above—we have to verify the nonexistence of arcs in the other direction. The number of dropped candidates from one branch, which is a threshold that allows detecting a fork, is adjusted dynamically, based on genome coverage in a given region of the graph and on the overall distribution of coverage in the whole data set.

As mentioned earlier, forks in the graph occur not only due to repetitive regions, but also due to errors and SNPs. Therefore, some additional checks have to be introduced to recognize the second type of forks. To ignore the second type of forks, which usually make contigs short, the algorithm compares the candidate sets from all branches. If they have a significant number of similar candidates, the fork is discarded.

### Contig correction

**Fig 6:**
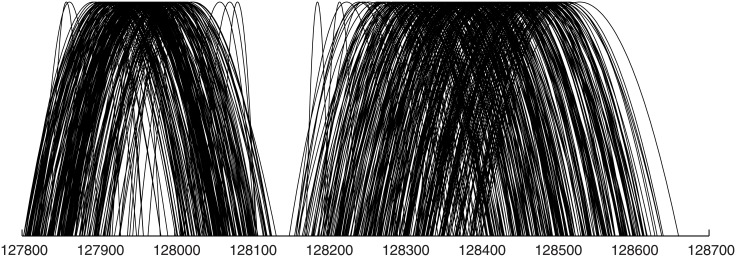
Contigs correction. Visualization of breaks in the continuity of paired-end information (shown as arches) on a real data set, mapped to a contig created by the traversal step.

**Fig 7:**
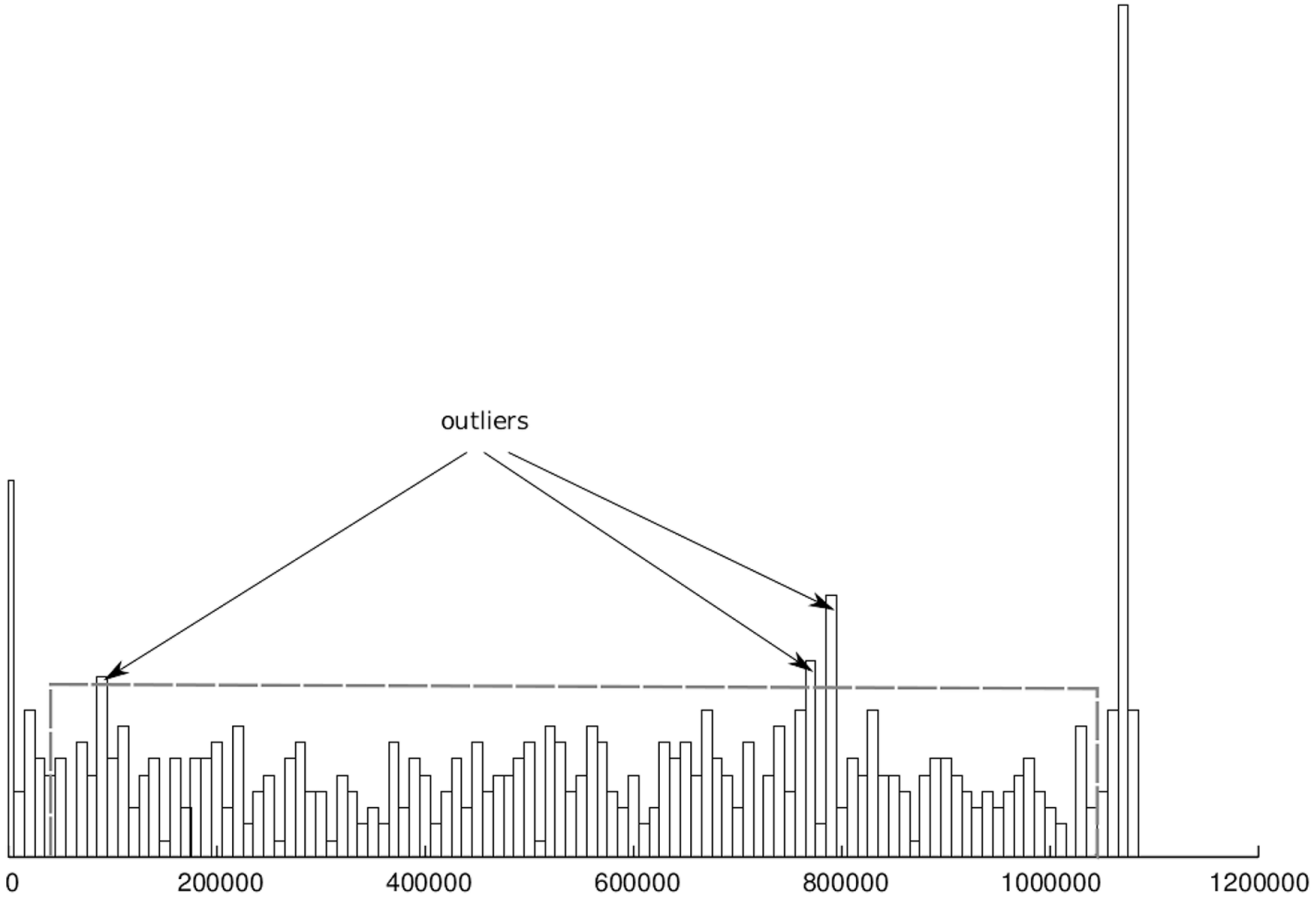
Contigs correction. Histogram visualizing the number of reads mapped to a given contig region and having the other read from the pair mapped to a different contig or a distant part of the same contig.

Although the fork detection step already identifies most of the alternative paths of the overlap graph, it operates only on the local context of reads, i.e., overlaps, and does not apply the information from paired-end sequencing. The contig correction step improves the quality of the contigs by cutting them at the point where the paired-end data suggest anomalies in overlaps. There are two main types of anomalies that can be detected:

(i) incoherence in paired-end reads continuity (Fig 6),
(ii) excessive density of paired-end reads pointing to different contigs or to distant parts of the same contig (Fig 7).

To find both types of anomalies, we developed a hyper-heuristic-driven algorithm. A hyper-heuristic method does not operate on the solution directly but uses simple procedures, called low-level heuristics, which modify a solution and are evaluated by a function. The algorithm can learn during the search process, whose procedure is better for a particular instance or at a particular time (more on hyper-heuristics can be found in [34–36]). Hyper-heuristics have already been successfully applied to some bioinformatics problems (e.g., [37–39]).

In our approach, a hyper-heuristic is a greedy algorithm, which operates on two low-level procedures. The first one detects the gap between paired-end reads that are consecutively mapped to a contig (anomaly (i)). The second one searches for pairs of reads, which are mapped within a far distance (anomaly (ii)).

The method is parameterized to adjust to different properties of the input data (e.g., low coverage or high insert size). Parameters allow defining the minimal continuity break width to be considered as an anomaly, set the threshold on the number of pairs of reads matched to different contigs to be considered as significant, or declare the minimum and maximum insert size in the data (see S1 Text for the reference to the complete list of the algorithm parameters). As the output of the hyper-heuristic method, we get a set of contig cuts that potentially reduce the misassembly rate.

### Contig trimming

When contigs are ready, the next step is to compose them into scaffolds. In contrast to the stage of contig correction, paired-end information is used here to merge contigs together instead of splitting them apart. To perform the scaffolding, we use external tools. The main assumption of the available scaffolding methods is that the input contigs do not overlap. GRASShopPER does not provide non-overlapping contigs out of the box. This is caused by the specific data abstraction of the OLC strategy. OLC does not operate on the nucleotide level, but instead wraps the reads in an additional layer of indirection. They become vertices with arcs representing overlaps. At this stage, one does not even need to load nucleic acid sequences of reads. However, there is a drawback—the exact position of the fork in the consensus alignment cannot be precisely indicated. Therefore, GRASShopPER contigs may not fulfill the expectations of the scaffolding methods. This is why there was a need to provide yet another step, contig trimming.

**Fig 8:**
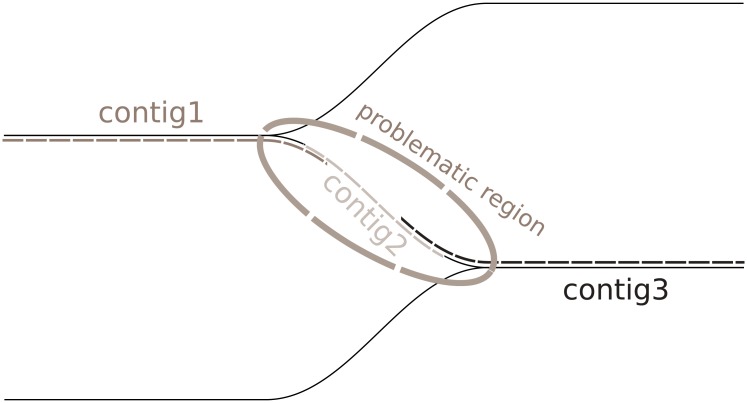
Visualization of the problem caused by closely located forks.

As overlaps in contigs produced by GRASShopPER are placed very close to the ends of the contigs, they are relatively easy to locate. However, one has to be extra cautious, because simply cutting them off from the contigs may bring undesirable consequences on the genome fraction metric. The problem is with possibly nearly located forks. It would lead to excessive scatter of contigs and loss of information from the shortest ones (see Fig 8). This is due to the fact that most of the scaffolding methods never take short contigs into account. Our approach attempts to minimize this effect.

The contig trimming method uses reads-to-contigs mapping to find repeated regions in the contigs. When it finds one, it performs the analysis whether a potential cutoff can cause genome fraction loss. If removing the region from one of the contigs shortens it too much, the method tries to cut the region off from the other considered contig. If also this one is not long enough, the contigs are left untouched to protect the valuable information.

## Supporting information

S1 Table Assemblies obtained for the data set Candidatus Microthrix parvicella strain Bio17-1. (DOCX)

S2 Table Assemblies obtained for the data set Caenorhabditis elegans strain N2. (DOCX)

S3 Table Assemblies obtained for the data set Homo sapiens chromosome 14. (DOCX)

S4 Table Scaffolding of the data set *Candidatus* Microthrix parvicella strain Bio17-1 for the assemblers GRASShopPER, SOAPdenovo2 and SGA with the combination of scaffolders SSPACE and SOAPdenovo2. (DOCX)

S5 Table Scaffolding of the data set *Caenorhabditis elegans* strain N2 for the assemblers GRASShopPER, SOAPdenovo2 and SGA with the combination of scaffolders SSPACE and SOAPdenovo2. (DOCX)

S6 Table Scaffolding of the data set *Homo sapiens* chromosome 14 for the assemblers GRASShopPER, SOAPdenovo2 and SGA with the combination of scaffolders SSPACE and SOAPdenovo2. (DOCX)

S1 Text Algorithm parameters. The list of GRASShopPER parameters. Description of how to run GRASShopPER. Parameters of all the methods used throughout computational experiment.
(DOCX)
