# Supplementary material for: GRASShopPER—An algorithm for de novo assembly based on GPU alignments
Source: PLoS One. 2018 Aug 16;13(8):e0202355. doi: 10.1371/journal.pone.0202355 (PMC6095601; doi:10.1371/journal.pone.0202355)
Supplement: S1 Table — (DOCX) [file pone.0202355.s001.docx]

**Table S1. Assemblies obtained for the data set *Candidatus* Microthrix parvicella strain Bio17-1 (metrics calculated by QUAST)**

| Genome statistics | GRASShopPER | Celera | Platanus | SGA | SOAPdenovo2 | Velvet | SPADES |
| --- | --- | --- | --- | --- | --- | --- | --- |
| Genome fraction (%) | 98.73 | 89.21 | 98.38 | 98.82 | 98.52 | 97.86 | 98.96 |
| Duplication ratio | 1.006 | 1.002 | 1.017 | 1.002 | 1.001 | 1.000 | 1.001 |
| Largest alignment | 126,696 | 50,960 | 25,116 | 101,782 | 107,154 | 166,835 | 740,450 |
| Total aligned length | 4,173,839 | 3,755,338 | 4,203,934 | 4,161,932 | 4,145,584 | 4,113,131 | 4,161,980 |
| NG50 | 33,570 | 11,255 | 5,286 | 32,697 | 34,653 | 78,563 | 156,137 |
| NG75 | 16,714 | 5,614 | 2,889 | 18,691 | 17,879 | 39,191 | 104,295 |
| NA50 | 33,566 | 12,191 | 5,281 | 32,697 | 34,804 | 77,856 | 150,746 |
| NA75 | 17,110 | 7,674 | 2,886 | 18,900 | 18,827 | 40,178 | 73,687 |
| NGA50 | 33,566 | 11,013 | 5,281 | 32,697 | 34,653 | 77,856 | 151,220 |
| NGA75 | 16,712 | 5,528 | 2,886 | 18,691 | 17,879 | 39,191 | 88,932 |
| LG50 | 38 | 110 | 238 | 39 | 37 | 19 | 9 |
| LG75 | 82 | 238 | 502 | 81 | 80 | 38 | 17 |
| LA50 | 38 | 91 | 239 | 38 | 36 | 20 | 10 |
| LA75 | 81 | 188 | 503 | 79 | 77 | 38 | 20 |
| LGA50 | 38 | 111 | 239 | 39 | 37 | 20 | 9 |
| LGA75 | 82 | 240 | 503 | 81 | 80 | 39 | 18 |
| # misassemblies | 4 | 4 | 1 | 3 | 1 | 6 | 5 |
| # relocations | 0 | 1 | 0 | 0 | 0 | 1 | 1 |
| # translocations | 4 | 3 | 1 | 3 | 1 | 4 | 4 |
| # inversions | 0 | 0 | 0 | 0 | 0 | 1 | 0 |
| # misassembled contigs | 4 | 4 | 1 | 3 | 1 | 6 | 5 |
| Misassembled contigs length | 10,783 | 31,643 | 5,697 | 36,678 | 31,008 | 329,627 | 369,639 |
| # local misassemblies | 6 | 11 | 0 | 0 | 0 | 6 | 2 |
| # unaligned mis. contigs | 0 | 0 | 0 | 0 | 0 | 0 | 0 |
| # fully unaligned contigs | 1 | 0 | 0 | 0 | 6 | 1 | 730 |
| Fully unaligned length | 400 | 0 | 0 | 0 | 2,196 | 1,282 | 233,715 |
| # partially unaligned contigs | 0 | 0 | 1 | 0 | 0 | 0 | 0 |
| Partially unaligned length | 0 | 0 | 654 | 0 | 0 | 0 | 0 |
| # mismatches | 343 | 214 | 20 | 44 | 23 | 809 | 66 |
| # indels | 73 | 38 | 9 | 13 | 1 | 401 | 11 |
| Indels length | 433 | 370 | 177 | 148 | 2 | 2,237 | 285 |
| # mismatches per 100 kbp | 8.27 | 5.71 | 0.48 | 1.06 | 0.56 | 19.67 | 1.59 |
| # indels per 100 kbp | 1.76 | 1.01 | 0.22 | 0.31 | 0.02 | 9.75 | 0.26 |
| # indels (≤ 5 bases) | 58 | 28 | 1 | 2 | 1 | 334 | 3 |
| # indels (> 5 bases) | 15 | 10 | 8 | 11 | 0 | 67 | 8 |
| no. contigs (> 0 bases) | 439 | 493 | 2107 | 668 | 949 | 103 | 1297 |
| no. contigs (≥250 bases) | 336 | 449 | 1395 | 257 | 267 | 103 | 808 |
| no. contigs (≥ 1 kb) | 254 | 424 | 966 | 215 | 220 | 103 | 64 |
| no. contigs (≥ 5 kb) | 159 | 256 | 257 | 161 | 157 | 88 | 49 |
| no. contigs (≥ 10 kb) | 112 | 127 | 66 | 118 | 115 | 75 | 44 |
| no. contigs (≥ 25 kb) | 57 | 21 | 1 | 56 | 54 | 51 | 30 |
| no. contigs (≥ 50 kb) | 19 | 1 | 0 | 20 | 21 | 29 | 23 |
| Largest contig | 126,700 | 50,960 | 25,116 | 101,782 | 107,154 | 166,835 | 740,450 |
| Total length | 4,176,754 | 3,755,980 | 4,204,730 | 4,162,092 | 4,147,902 | 4,114,665 | 4,396,132 |
| Total length (> 0 bases) | 4,191,347 | 3,763,334 | 4,292,086 | 4,217,042 | 4,209,976 | 4,114,665 | 4,495,722 |
| Total length (≥ 1 kb) | 4,134,542 | 3,741,901 | 3,982,304 | 4,143,129 | 4,125,178 | 4,114,665 | 4,161,012 |
| Total length (≥ 5 kb) | 3,899,672 | 3,249,038 | 2,199,348 | 4,024,384 | 3,971,923 | 4,086,977 | 4,130,417 |
| Total length (≥ 10 kb) | 3,555,679 | 2,283,432 | 902,318 | 3,703,730 | 3,647,186 | 3,991,452 | 4,096,520 |
| Total length (≥ 25 kb) | 2,643,497 | 675,660 | 25,116 | 2,612,353 | 2,606,551 | 3,569,592 | 3,877,248 |
| Total length (≥ 50 kb) | 1,366,887 | 50,960 | 0 | 1,387,790 | 1,454,663 | 2,762,754 | 3,608,676 |
| N50 | 33,570 | 12,191 | 5,286 | 32,697 | 34,804 | 78,563 | 151,220 |
| N75 | 17,110 | 7,733 | 2,886 | 18,900 | 18,827 | 40,178 | 82,660 |
| L50 | 38 | 91 | 239 | 38 | 36 | 19 | 10 |
| L75 | 81 | 187 | 503 | 79 | 77 | 37 | 19 |
| GC (%) | 66.40 | 66.46 | 66.48 | 66.41 | 66.42 | 66.43 | 65.87 |
| Similarity statistics |  |  |  |  |  |  |  |
| # similar correct contigs | 5 | 4 | 0 | 9 | 9 | 10 | 7 |
| # similar misassembled blocks | 0 | 0 | 0 | 0 | 0 | 0 | 0 |
