# Supplementary material for: GRASShopPER—An algorithm for de novo assembly based on GPU alignments
Source: PLoS One. 2018 Aug 16;13(8):e0202355. doi: 10.1371/journal.pone.0202355 (PMC6095601; doi:10.1371/journal.pone.0202355)
Supplement: S2 Table — (DOCX) [file pone.0202355.s002.docx]

**Table S2. Assemblies obtained for the data set *Caenorhabditis elegans* strain N2 (metrics calculated by QUAST)**

| Genome statistics | GRASShopPER | Celera | Platanus | SGA | SOAPdenovo2 | Velvet | SPADES |
| --- | --- | --- | --- | --- | --- | --- | --- |
| Genome fraction (%) | 95.47 | 78.81 | 88.39 | 93.92 | 92.58 | 85.61 | 94.81 |
| Duplication ratio | 1.019 | 1.020 | 1.004 | 1.008 | 1.004 | 1.004 | 1.004 |
| Largest alignment | 96,261 | 33,627 | 63,884 | 80,404 | 83,885 | 58,073 | 180,696 |
| Total aligned length | 97,504,779 | 80,514,762 | 88,972,062 | 94,936,888 | 93,192,365 | 85,981,341 | 95,338,850 |
| NG50 | 7,772 | 3,982 | 4,157 | 6,618 | 6,364 | 7,000 | 20,063 |
| NG75 | 2,793 | 1,789 | 1,402 | 2,665 | 2,486 | 3,018 | 8,732 |
| NA50 | 8,119 | 4,977 | 4,760 | 6,767 | 6,616 | 7,730 | 18,706 |
| NA75 | 3,090 | 2,945 | 1,791 | 2,733 | 2,606 | 3,720 | 7,615 |
| NGA50 | 7,771 | 3,903 | 4,088 | 6,581 | 6,313 | 6,736 | 18,679 |
| NGA75 | 2,783 | 1,700 | 1,277 | 2,557 | 2,325 | 2,576 | 7,495 |
| LG50 | 3,135 | 6,958 | 5,403 | 3,770 | 3,817 | 3,918 | 1,333 |
| LG75 | 8,520 | 16,142 | 15,960 | 9,784 | 10,188 | 9,328 | 3,214 |
| LA50 | 2,975 | 4,883 | 4,533 | 3,644 | 3,594 | 3,303 | 1,390 |
| LA75 | 7,879 | 10,233 | 12,580 | 9,394 | 9,487 | 7,487 | 3,452 |
| LGA50 | 3,136 | 7,045 | 5,437 | 3,781 | 3,828 | 4,001 | 1,400 |
| LGA75 | 8,533 | 16,497 | 16,533 | 9,911 | 10,409 | 9,917 | 3,490 |
| # misassemblies | 142 | 537 | 5 | 55 | 12 | 342 | 505 |
| # relocations | 55 | 242 | 5 | 24 | 8 | 197 | 203 |
| # translocations | 70 | 265 | 0 | 22 | 4 | 103 | 286 |
| # inversions | 17 | 30 | 0 | 9 | 0 | 42 | 16 |
| # misassembled contigs | 140 | 524 | 5 | 55 | 12 | 337 | 475 |
| Misassembled contigs length | 176,244 | 2,402,183 | 47,094 | 215,464 | 57,586 | 2,228,569 | 8,519,276 |
| # local misassemblies | 375 | 359 | 16 | 74 | 34 | 1,080 | 468 |
| # unaligned mis. contigs | 6 | 3 | 0 | 10 | 0 | 9 | 6 |
| # fully unaligned contigs | 316 | 359 | 3,385 | 3,381 | 3,664 | 1,297 | 1,012 |
| Fully unaligned length | 110,312 | 622,935 | 3,325,663 | 3,461,995 | 4,034,428 | 3,809,026 | 3,844,786 |
| # partially unaligned contigs | 26 | 35 | 3 | 30 | 10 | 194 | 156 |
| Partially unaligned length | 23,584 | 36,977 | 2,555 | 30,437 | 9,151 | 219,312 | 604,774 |
| # mismatches | 26,639 | 22,294 | 1,261 | 3,654 | 1,280 | 42,507 | 16,471 |
| # indels | 5,710 | 4,751 | 814 | 1,444 | 973 | 20,297 | 5,107 |
| Indels length | 24,537 | 23,434 | 1,613 | 6,826 | 2,055 | 107,012 | 35,318 |
| # mismatches per 100 kbp | 27.82 | 28.21 | 1.42 | 3.88 | 1.38 | 49.51 | 17.32 |
| # indels per 100 kbp | 5.96 | 6.01 | 0.92 | 1.53 | 1.05 | 23.64 | 5.37 |
| # indels (≤ 5 bases) | 5,098 | 4,004 | 785 | 1,227 | 935 | 16,121 | 3,758 |
| # indels (> 5 bases) | 612 | 747 | 29 | 217 | 38 | 4,176 | 1,349 |
| no. contigs (>0 bases) | 82,283 | 21,503 | 233,557 | 150,360 | 160,015 | 17,510 | 52,752 |
| no. contigs (≥ 250 bases) | 38,336 | 20,766 | 42,224 | 34,185 | 33,847 | 17,510 | 13,779 |
| no. contigs (≥ 1 kb) | 15,971 | 20,220 | 20,742 | 18,911 | 19,006 | 17,510 | 9,320 |
| no. contigs (≥ 5 kb) | 5,108 | 4,912 | 4,328 | 5,246 | 5,100 | 5,897 | 4,915 |
| no. contigs (≥ 10 kb) | 2,247 | 1,108 | 1,572 | 2,122 | 2,004 | 2,278 | 2,866 |
| no. contigs (≥ 25 kb) | 401 | 17 | 167 | 287 | 307 | 202 | 946 |
| no. contigs (≥ 50 kb) | 39 | 0 | 6 | 14 | 17 | 5 | 244 |
| Largest contig | 96,261 | 33,627 | 63,884 | 80,404 | 83,885 | 58,073 | 180,696 |
| Total length | 97,732,475 | 81,266,258 | 92,311,186 | 98,462,410 | 97,258,190 | 90,225,299 | 99,896,951 |
| Total length (> 0 bases) | 105,342,642 | 81,371,411 | 106,058,593 | 112,106,195 | 107,344,742 | 90,225,299 | 104,090,309 |
| Total length (≥ 1 kb) | 88,117,765 | 80,949,133 | 80,896,148 | 90,499,368 | 89,427,285 | 90,225,299 | 97,786,683 |
| Total length (≥ 5 kb) | 62,424,381 | 41,036,632 | 45,262,686 | 58,644,040 | 57,359,108 | 61,870,406 | 86,513,734 |
| Total length (≥ 10 kb) | 42,323,674 | 15,057,871 | 26,052,975 | 36,834,573 | 35,720,450 | 36,489,743 | 71,974,788 |
| Total length (≥ 25 kb) | 14,422,596 | 463,790 | 5,383,571 | 9,690,975 | 10,430,798 | 6,314,356 | 41,514,017 |
| Total length (≥ 50 kb) | 2,369,995 | 0 | 341,280 | 833,951 | 1,003,425 | 267,395 | 17,250,995 |
| N50 | 8,123 | 5,048 | 4,821 | 6,802 | 6,675 | 8,011 | 20,138 |
| N75 | 3,099 | 3,010 | 1,901 | 2,827 | 2,749 | 4,081 | 8,842 |
| L50 | 2,974 | 4,832 | 4,510 | 3,634 | 3,584 | 3,246 | 1,323 |
| L75 | 7,868 | 10,084 | 12,305 | 9,286 | 9,319 | 7,184 | 3,181 |
| GC (%) | 35.39 | 34.67 | 35.57 | 35.60 | 35.81 | 35.87 | 35.92 |
| # similar correct contigs | 233 | 81 | 292 | 318 | 329 | 183 | 222 |
| # similar misassembled blocks | 0 | 0 | 0 | 0 | 0 | 0 | 3 |
