# Supplementary material for: GRASShopPER—An algorithm for de novo assembly based on GPU alignments
Source: PLoS One. 2018 Aug 16;13(8):e0202355. doi: 10.1371/journal.pone.0202355 (PMC6095601; doi:10.1371/journal.pone.0202355)
Supplement: S3 Table — (DOCX) [file pone.0202355.s003.docx]

**Table S3. Assemblies obtained for the data set *Homo sapiens* chromosome 14 (metrics calculated by QUAST)**

| Genome statistics | GRASShopPER | Celera | Platanus | SGA | SOAPdenovo2 | Velvet | SPADES |
| --- | --- | --- | --- | --- | --- | --- | --- |
| Genome fraction (%) | 92.28 | 75.96 | 71.80 | 88.30 | 88.99 | 72.33 | 93.53 |
| Duplication ratio | 1.038 | 1.005 | 1.005 | 1.007 | 1.007 | 1.007 | 1.011 |
| Largest alignment | 38,022 | 39,634 | 13,122 | 30,294 | 28,332 | 41,564 | 58,597 |
| Total aligned length | 86,648,379 | 69,025,598 | 65,344,557 | 80,527,423 | 81,146,875 | 65,606,013 | 85,602,007 |
| NG50 | 2,500 | 3,032 | 782 | 2,914 | 2,421 | 2,801 | 4,878 |
| NG75 | 1,020 | 1,154 | - | 1,207 | 1,000 | - | 2,260 |
| NA50 | 2,665 | 3,934 | 1,216 | 3,378 | 2,797 | 3,891 | 5,088 |
| NA75 | 1,196 | 2,361 | 670 | 1,729 | 1,398 | 2,153 | 2,563 |
| NGA50 | 2,500 | 2,891 | 782 | 2,909 | 2,418 | 2,628 | 4,755 |
| NGA75 | 1,014 | 1,077 | - | 1,202 | 997 | - | 2,204 |
| LG50 | 9,417 | 8,209 | 28,602 | 8,399 | 9,847 | 8,232 | 4,981 |
| LG75 | 23,358 | 19,730 | - | 20,283 | 24,232 | - | 11,782 |
| LA50 | 8,723 | 5,363 | 15,668 | 6,817 | 8,061 | 4,961 | 4,606 |
| LA75 | 20,918 | 11,073 | 33,769 | 15,167 | 18,347 | 10,802 | 10,556 |
| LGA50 | 9,435 | 8,541 | 28,606 | 8,411 | 9,856 | 8,591 | 5,097 |
| LGA75 | 23,419 | 20,683 | - | 20,329 | 24,264 | - | 12,081 |
| # misassemblies | 125 | 1,145 | 0 | 51 | 17 | 376 | 559 |
| # relocations | 119 | 1,132 | 0 | 50 | 16 | 372 | 553 |
| # translocations | 0 | 0 | 0 | 0 | 0 | 0 | 0 |
| # inversions | 6 | 13 | 0 | 1 | 1 | 4 | 6 |
| # misassembled contigs | 123 | 1,110 | 0 | 51 | 17 | 358 | 544 |
| Misassembled contigs length | 206,606 | 5,039,195 | 0 | 171,303 | 92,022 | 1,606,153 | 3,601,034 |
| # local misassemblies | 247 | 54 | 3 | 12 | 7 | 1,627 | 101 |
| # unaligned mis. contigs | 0 | 1 | 0 | 0 | 0 | 208 | 0 |
| # fully unaligned contigs | 292 | 11 | 35 | 68 | 119 | 117 | 96 |
| Fully unaligned length | 114,667 | 20,422 | 14,936 | 48,602 | 63,202 | 167,443 | 59,738 |
| # partially unaligned contigs | 10 | 58 | 2 | 7 | 3 | 894 | 33 |
| Partially unaligned length | 9,791 | 59,437 | 1,075 | 5,017 | 1,655 | 1,281,739 | 28,452 |
| # mismatches | 119,141 | 93,952 | 47,541 | 66,176 | 66,162 | 88,381 | 88,886 |
| # indels | 15,965 | 8,395 | 1,723 | 6,324 | 7,350 | 20,317 | 15,673 |
| Indels length | 44,922 | 29,341 | 4,792 | 21,999 | 21,830 | 113,974 | 56,603 |
| # mismatches per 100 kbp | 142.56 | 136.56 | 73.11 | 82.75 | 82.09 | 134.91 | 104.94 |
| # indels per 100 kbp | 19.10 | 12.20 | 2.65 | 7.91 | 9.12 | 31.01 | 18.50 |
| # indels (≤ 5 bases) | 14,587 | 7,213 | 1,581 | 5,458 | 6,523 | 16,005 | 13,367 |
| # indels (> 5 bases) | 1,378 | 1,182 | 142 | 866 | 827 | 4,312 | 2,306 |
| no. contigs (> 0 bases) | 81,314 | 21,003 | 574,441 | 97,520 | 239,297 | 21,153 | 62,461 |
| no. contigs (≥ 250 bases) | 64,638 | 20,930 | 72,766 | 40,353 | 48,947 | 21,153 | 29,935 |
| no. contigs (≥ 1 kb) | 24,310 | 20,880 | 21,035 | 22,930 | 24,237 | 21,153 | 19,521 |
| no. contigs (≥ 5 kb) | 2,988 | 3,607 | 259 | 3,460 | 2,830 | 3,398 | 4,796 |
| no. contigs (≥ 10 kb) | 468 | 559 | 1 | 525 | 390 | 651 | 1,354 |
| no. contigs (≥ 25 kb) | 7 | 4 | 0 | 12 | 1 | 10 | 105 |
| no. contigs (≥ 50 kb) | 0 | 0 | 0 | 0 | 0 | 0 | 1 |
| Largest contig | 38,050 | 39,634 | 13,122 | 30,294 | 28,332 | 41,883 | 58,597 |
| Total length | 86,896,666 | 69,199,589 | 65,363,965 | 80,596,325 | 81,225,918 | 67,391,861 | 85,737,619 |
| Total length (> 0 bases) | 89,502,184 | 69,207,400 | 94,167,749 | 87,432,262 | 96,466,017 | 67,391,861 | 90,306,487 |
| Total length (≥ 1 kb) | 68,882,304 | 69,170,881 | 38,597,351 | 70,834,705 | 67,932,568 | 67,391,861 | 80,023,810 |
| Total length (≥ 5 kb) | 22,908,025 | 27,526,267 | 1,572,451 | 26,600,652 | 21,391,335 | 27,404,895 | 44,374,275 |
| Total length (≥ 10 kb) | 5,994,088 | 7,209,319 | 13,122 | 6,914,656 | 5,040,504 | 8,827,312 | 20,666,068 |
| Total length (≥ 25 kb) | 214,072 | 129,791 | 0 | 330,519 | 28,332 | 299,270 | 3,245,421 |
| Total length (≥ 50 kb) | 0 | 0 | 0 | 0 | 0 | 0 | 58,597 |
| N50 | 2,673 | 4,108 | 1,217 | 3,382 | 2,799 | 4,061 | 5,234 |
| N75 | 1,200 | 2,478 | 671 | 1,734 | 1,399 | 2,318 | 2,626 |
| L50 | 8,706 | 5,173 | 15,667 | 6,808 | 8,054 | 4,797 | 4,502 |
| L75 | 20,868 | 10,623 | 33,763 | 15,138 | 18,328 | 10,296 | 10,296 |
| GC (%) | 40.36 | 39.31 | 38.76 | 40.16 | 40.26 | 39.63 | 40.63 |
| # similar correct contigs | 63 | 28 | 0 | 47 | 55 | 31 | 30 |
| # similar misassembled blocks | 0 | 0 | 0 | 0 | 0 | 0 | 0 |
