# Supplementary material for: GRASShopPER—An algorithm for de novo assembly based on GPU alignments
Source: PLoS One. 2018 Aug 16;13(8):e0202355. doi: 10.1371/journal.pone.0202355 (PMC6095601; doi:10.1371/journal.pone.0202355)
Supplement: S4 Table — (DOCX) [file pone.0202355.s004.docx]

**Table S4. Scaffolding of the data set *Candidatus* Microthrix parvicella strain Bio17-1 for the assemblers GRASShopPER, SOAPdenovo2 and SGA with the combination of scaffolders SSPACE and SOAPdenovo2 (metrics calculated by QUAST)**

| Assembler | GRASShopPER | GRASShopPER | GRASShopPER | GRASShopPER | GRASShopPER | GRASShopPER | SGA | SGA | SOAPdenovo2 | SOAPdenovo2 |
| --- | --- | --- | --- | --- | --- | --- | --- | --- | --- | --- |
| Scaffolder | - | - | SSPACE | SSPACE | SOAPdenovo2 | SOAPdenovo2 | SSPACE | SOAPdenovo2 | SSPACE | SOAPdenovo2 |
| Postprocessing | no | yes | no | yes | no | yes |  |  |  |  |
| Genome fraction (%) | 98.73 | 98.602 | 98.803 | 98.602 | 98.8 | 98.613 | 98.819 | 98.819 | 98.522 | 98.522 |
| Duplication ratio | 1.006 | 1.006 | 1.007 | 1.006 | 1.007 | 1.006 | 1.002 | 1.002 | 1.001 | 1.001 |
| Largest alignment | 126,696 | 126,696 | 128,161 | 126,696 | 128,161 | 126,696 | 101,782 | 101,782 | 107,154 | 107,154 |
| Total aligned length | 4,173,839 | 4,165,836 | 4,179,084 | 4,165,836 | 4,179,084 | 4,166,233 | 4,161,932 | 4,161,932 | 4,145,584 | 4,145,584 |
| NG50 | 33,570 | 33,570 | 36,887 | 33,570 | 36,887 | 33,570 | 32,697 | 32,697 | 34,653 | 34,653 |
| NG75 | 16,714 | 16,714 | 20,903 | 16,714 | 20,903 | 16,714 | 18,691 | 18,691 | 17,879 | 17,879 |
| NA50 | 33,566 | 33,566 | 36,884 | 33,566 | 36,884 | 33,566 | 32,697 | 32,697 | 34,804 | 34,804 |
| NA75 | 17,110 | 17,110 | 19,217 | 17,110 | 19,217 | 17,110 | 18,900 | 18,900 | 18,827 | 18,827 |
| NGA50 | 33,566 | 33,566 | 36,514 | 33,566 | 36,514 | 33,566 | 32,697 | 32,697 | 34,653 | 34,653 |
| NGA75 | 16,712 | 16,712 | 18,651 | 16,712 | 18,651 | 16,712 | 18,691 | 18,691 | 17,879 | 17,879 |
| LG50 | 38 | 38 | 32 | 38 | 32 | 38 | 39 | 39 | 37 | 37 |
| LG75 | 82 | 82 | 69 | 82 | 69 | 82 | 81 | 81 | 80 | 80 |
| LA50 | 38 | 38 | 32 | 38 | 32 | 38 | 38 | 38 | 36 | 36 |
| LA75 | 81 | 81 | 71 | 81 | 71 | 81 | 79 | 79 | 77 | 77 |
| LGA50 | 38 | 38 | 33 | 38 | 33 | 38 | 39 | 39 | 37 | 37 |
| LGA75 | 82 | 82 | 72 | 82 | 72 | 82 | 81 | 81 | 80 | 80 |
| Misassemblies |  |  |  |  |  |  |  |  |  |  |
| # misassemblies | 4 | 4 | 25 | 4 | 25 | 5 | 3 | 3 | 1 | 1 |
| # relocations | 0 | 0 | 11 | 0 | 11 | 1 | 0 | 0 | 0 | 0 |
| # translocations | 4 | 4 | 13 | 4 | 13 | 4 | 3 | 3 | 1 | 1 |
| # inversions | 0 | 0 | 1 | 0 | 1 | 0 | 0 | 0 | 0 | 0 |
| # misassembled contigs | 4 | 4 | 20 | 4 | 20 | 5 | 3 | 3 | 1 | 1 |
| Misassembled contigs length | 10,783 | 10,671 | 476,019 | 10,671 | 476,019 | 53,130 | 36,678 | 36,678 | 31,008 | 31,008 |
| # local misassemblies | 6 | 6 | 14 | 6 | 14 | 12 | 0 | 0 | 0 | 0 |
| # unaligned mis. contigs | 0 | 0 | 0 | 0 | 0 | 0 | 0 | 0 | 0 | 0 |
| Unaligned |  |  |  |  |  |  |  |  |  |  |
| # fully unaligned contigs | 1 | 1 | 1 | 1 | 1 | 0 | 0 | 0 | 6 | 6 |
| Fully unaligned length | 400 | 360 | 400 | 360 | 400 | 0 | 0 | 0 | 2196 | 2196 |
| # partially unaligned contigs | 0 | 0 | 0 | 0 | 0 | 0 | 0 | 0 | 0 | 0 |
| Partially unaligned length | 0 | 0 | 0 | 0 | 0 | 0 | 0 | 0 | 0 | 0 |
| Mismatches |  |  |  |  |  |  |  |  |  |  |
| # mismatches | 343 | 312 | 420 | 312 | 416 | 327 | 44 | 44 | 23 | 23 |
| # indels | 73 | 65 | 73 | 65 | 73 | 74 | 13 | 13 | 1 | 1 |
| Indels length | 433 | 344 | 518 | 344 | 518 | 410 | 148 | 148 | 2 | 2 |
| # mismatches per 100 kb | 8.27 | 7.53 | 10.11 | 7.53 | 10.02 | 7.89 | 1.06 | 1.06 | 0.56 | 0.56 |
| # indels per 100 kb | 1.76 | 1.57 | 1.76 | 1.57 | 1.76 | 1.79 | 0.31 | 0.31 | 0.02 | 0.02 |
| # indels (≤ 5 bases) | 58 | 51 | 57 | 51 | 57 | 55 | 2 | 2 | 1 | 1 |
| # indels (> 5 bases) | 15 | 14 | 16 | 14 | 16 | 19 | 11 | 11 | 0 | 0 |
| # N's | 0 | 0 | 0 | 0 | 0 | 35 | 0 | 0 | 0 | 0 |
| # N's per 100 kb | 0 | 0 | 0 | 0 | 0 | 0.84 | 0 | 0 | 0 | 0 |
| Statistics without reference |  |  |  |  |  |  |  |  |  |  |
| no. contigs (> 0 bases) | 439 | 439 | 314 | 439 | 314 | 427 | 668 | 658 | 949 | 500 |
| no. contigs (≥250 bases) | 336 | 335 | 261 | 335 | 261 | 328 | 257 | 257 | 267 | 267 |
| no. contigs (≥ 1 kb) | 254 | 253 | 213 | 253 | 213 | 250 | 215 | 215 | 220 | 220 |
| no. contigs (≥ 5 kb) | 159 | 159 | 144 | 159 | 144 | 159 | 161 | 161 | 157 | 157 |
| no. contigs (≥ 10 kb) | 112 | 111 | 105 | 111 | 105 | 111 | 118 | 118 | 115 | 115 |
| no. contigs (≥ 25 kb) | 57 | 56 | 54 | 56 | 54 | 56 | 56 | 56 | 54 | 54 |
| no. contigs (≥ 50 kb) | 19 | 19 | 24 | 19 | 24 | 19 | 20 | 20 | 21 | 21 |
| Largest contig | 126,700 | 126,700 | 143,713 | 126,700 | 143,713 | 126,700 | 101,782 | 101,782 | 107,154 | 107,154 |
| Total length | 4,176,754 | 4,168,184 | 4,182,212 | 4,168,184 | 4,182,212 | 4,168,263 | 4,162,092 | 4,162,092 | 4,147,902 | 4,147,902 |
| Total length (> 0 bases) | 4,191,347 | 4,183,017 | 4,191,347 | 4,183,017 | 4,191,347 | 4,182,524 | 4,217,042 | 4,216,092 | 4,209,976 | 4,178,931 |
| Total length (≥ 1 kb) | 4,134,542 | 4,127,919 | 4,157,138 | 4,127,919 | 4,157,138 | 4,130,441 | 4,143,129 | 4,143,129 | 4,125,178 | 4,125,178 |
| Total length (≥ 5 kb) | 3,899,672 | 3,896,671 | 3,985,581 | 3,896,671 | 3,985,581 | 3,907,819 | 4,024,384 | 4,024,384 | 3,971,923 | 3,971,923 |
| Total length (≥ 10 kb) | 3,555,679 | 3,543,792 | 3,687,441 | 3,543,792 | 3,687,441 | 3,552,561 | 3,703,730 | 3,703,730 | 3,647,186 | 3,647,186 |
| Total length (≥ 25 kb) | 2,643,497 | 2,617,705 | 2,816,700 | 2,617,705 | 2,816,700 | 2,618,860 | 2,612,353 | 2,612,353 | 2,606,551 | 2,606,551 |
| Total length (≥ 50 kb) | 1,366,887 | 1,366,651 | 1,782,230 | 1,366,651 | 1,782,230 | 1,366,651 | 1,387,790 | 1,387,790 | 1,454,663 | 1,454,663 |
| N50 | 33,570 | 33,570 | 42,487 | 33,570 | 42,487 | 33,570 | 32,697 | 32,697 | 34,804 | 34,804 |
| N75 | 17,110 | 17,110 | 20,903 | 17,110 | 20,903 | 17,110 | 18,900 | 18,900 | 18,827 | 18,827 |
| L50 | 38 | 38 | 31 | 38 | 31 | 38 | 38 | 38 | 36 | 36 |
| L75 | 81 | 81 | 69 | 81 | 69 | 81 | 79 | 79 | 77 | 77 |
| GC (%) | 66.40 | 66.41 | 66.40 | 66.41 | 66.40 | 66.41 | 66.41 | 66.41 | 66.42 | 66.42 |
