# Supplementary material for: GRASShopPER—An algorithm for de novo assembly based on GPU alignments
Source: PLoS One. 2018 Aug 16;13(8):e0202355. doi: 10.1371/journal.pone.0202355 (PMC6095601; doi:10.1371/journal.pone.0202355)
Supplement: S5 Table — (DOCX) [file pone.0202355.s005.docx]

**Table S5. Scaffolding of the data set *Caenorhabditis elegans* strain N2 for the assemblers GRASShopPER, SOAPdenovo2 and SGA with the combination of scaffolders SSPACE and SOAPdenovo2 (metrics calculated by QUAST)**

| Assembler | GRASShopPER | GRASShopPER | GRASShopPER | GRASShopPER | GRASShopPER | GRASShopPER | SGA | SGA | SOAPdenovo2 | SOAPdenovo2 |
| --- | --- | --- | --- | --- | --- | --- | --- | --- | --- | --- |
| Scaffolder | - | - | SSPACE | SSPACE | SOAPdenovo2 | SOAPdenovo2 | SSPACE | SOAPdenovo2 | SSPACE | SOAPdenovo2 |
| Postprocessing | no | yes | no | yes | no | yes |  |  |  |  |
| Genome fraction (%) | 95.47 | 95.45 | 96.463 | 96.328 | 95.748 | 95.357 | 94.981 | 94.037 | 93.549 | 92.602 |
| Duplication ratio | 1.019 | 1.019 | 1.033 | 1.033 | 1.022 | 1.021 | 1.017 | 1.01 | 1.002 | 1.005 |
| Largest alignment | 96,261 | 96,261 | 191,328 | 191,329 | 126,145 | 126,121 | 235,364 | 105,248 | 119,149 | 126,063 |
| Total aligned length | 97,504,779 | 97,471,748 | 99,674,412 | 99,471,971 | 97,919,150 | 97,363,265 | 96,654,453 | 94,990,734 | 93,929,080 | 93,040,462 |
| NG50 | 7772 | 7772 | 18,453 | 15,258 | 11,752 | 11,352 | 13,475 | 10,187 | 12,859 | 12,023 |
| NG75 | 2793 | 2791 | 7737 | 6080 | 4528 | 4306 | 5575 | 4149 | 4920 | 4932 |
| NA50 | 8119 | 8125 | 17,558 | 15,072 | 11,864 | 11,632 | 13,280 | 10,319 | 13,070 | 12,066 |
| NA75 | 3090 | 3095 | 7218 | 5909 | 4690 | 4563 | 5266 | 4144 | 4917 | 4845 |
| NGA50 | 7771 | 7771 | 17,566 | 15,034 | 11,510 | 11,244 | 13,321 | 10,105 | 12,627 | 11,749 |
| NGA75 | 2783 | 2783 | 7225 | 5878 | 4408 | 4240 | 5330 | 3951 | 4554 | 4591 |
| LG50 | 3135 | 3135 | 1430 | 1676 | 2196 | 2250 | 1898 | 2530 | 1957 | 2166 |
| LG75 | 8520 | 8522 | 3523 | 4286 | 5660 | 5844 | 4804 | 6421 | 5153 | 5472 |
| LA50 | 2975 | 2973 | 1489 | 1691 | 2149 | 2172 | 1933 | 2482 | 1892 | 2126 |
| LA75 | 7879 | 7871 | 3701 | 4347 | 5473 | 5558 | 4949 | 6299 | 4980 | 5376 |
| LGA50 | 3136 | 3136 | 1488 | 1698 | 2226 | 2260 | 1922 | 2546 | 1971 | 2189 |
| LGA75 | 8533 | 8535 | 3698 | 4372 | 5770 | 5901 | 4906 | 6537 | 5302 | 5618 |
| Misassemblies |  |  |  |  |  |  |  |  |  |  |
| # misassemblies | 142 | 137 | 1290 | 915 | 773 | 496 | 586 | 522 | 95 | 172 |
| # relocations | 55 | 53 | 587 | 392 | 339 | 192 | 272 | 188 | 63 | 89 |
| # translocations | 70 | 67 | 611 | 455 | 385 | 268 | 263 | 309 | 18 | 74 |
| # inversions | 17 | 17 | 92 | 68 | 49 | 36 | 51 | 25 | 14 | 9 |
| # misassembled contigs | 140 | 135 | 1170 | 828 | 729 | 461 | 510 | 443 | 95 | 159 |
| Misassembled contigs length | 176,244 | 174,020 | 10,649,367 | 4,798,405 | 3,599,227 | 1,231,432 | 2,756,593 | 1,229,978 | 838,178 | 1,499,912 |
| # local misassemblies | 375 | 363 | 13652 | 13,755 | 816 | 830 | 8145 | 631 | 154 | 764 |
| # unaligned mis. contigs | 6 | 4 | 54 | 53 | 73 | 107 | 48 | 103 | 2 | 80 |
| Unaligned |  |  |  |  |  |  |  |  |  |  |
| # fully unaligned contigs | 316 | 324 | 446 | 477 | 417 | 526 | 2811 | 2720 | 2374 | 3665 |
| Fully unaligned length | 110,312 | 109,778 | 188,945 | 194,679 | 174,557 | 267,107 | 3,591,711 | 3,575,024 | 4,241,410 | 4,811,364 |
| # partially unaligned contigs | 26 | 26 | 196 | 172 | 167 | 350 | 131 | 215 | 37 | 457 |
| Partially unaligned length | 23,584 | 22,874 | 166,517 | 145,669 | 144,253 | 354,863 | 143,872 | 220,855 | 40,045 | 627,795 |
| Mismatches |  |  |  |  |  |  |  |  |  |  |
| # mismatches | 26,634 | 26,229 | 38,368 | 33,770 | 33,061 | 28,750 | 12,197 | 7359 | 3774 | 3085 |
| # indels | 5710 | 5615 | 14,759 | 18,635 | 11,288 | 17,316 | 13,106 | 11,988 | 9051 | 18,323 |
| Indels length | 24,537 | 23,525 | 337,091 | 346,978 | 176,756 | 308,121 | 437,253 | 365,498 | 116,189 | 446,650 |
| # mismatches per 100 kb | 27.82 | 27.4 | 39.66 | 34.96 | 34.43 | 30.06 | 12.8 | 7.8 | 4.02 | 3.32 |
| # indels per 100 kb | 5.96 | 5.87 | 15.26 | 19.29 | 11.76 | 18.11 | 13.76 | 12.71 | 9.65 | 19.73 |
| # indels (≤ 5 bases) | 5098 | 5032 | 8455 | 12,205 | 6600 | 6807 | 4225 | 2668 | 3502 | 3428 |
| # indels (> 5 bases) | 612 | 583 | 6304 | 6430 | 4688 | 10,509 | 8881 | 9320 | 5549 | 14,895 |
| # N's | 0 | 0 | 21,401 | 26,984 | 69,762 | 229,678 | 29,030 | 149,173 | 12,021 | 400,430 |
| # N's per 100 kb | 0 | 0 | 21.33 | 26.96 | 70.84 | 233.71 | 28.86 | 150.68 | 12.23 | 405.42 |
| Statistics without reference |  |  |  |  |  |  |  |  |  |  |
| no. contigs (> 0 bases) | 82,283 | 82,283 | 47,290 | 52,277 | 66,487 | 68,787 | 123,022 | 129,145 | 137,704 | 39,909 |
| no. contigs (≥250 bases) | 38,336 | 38,336 | 18,206 | 21,371 | 27,602 | 28,586 | 20,738 | 24,959 | 19,839 | 20,248 |
| no. contigs (≥ 1 kb) | 15,971 | 15,967 | 10,021 | 11,677 | 12,955 | 13,128 | 13,025 | 14,944 | 13,201 | 13,654 |
| no. contigs (≥ 5 kb) | 5108 | 5108 | 4857 | 4973 | 5170 | 5133 | 5272 | 5432 | 5070 | 5405 |
| no. contigs (≥ 10 kb) | 2247 | 2247 | 2824 | 2702 | 2637 | 2605 | 2724 | 2600 | 2602 | 2692 |
| no. contigs (≥ 25 kb) | 401 | 401 | 895 | 780 | 624 | 611 | 729 | 517 | 700 | 628 |
| no. contigs (≥ 50 kb) | 39 | 39 | 225 | 183 | 90 | 88 | 131 | 69 | 136 | 89 |
| Largest contig | 96,261 | 96,261 | 191,334 | 191,335 | 126,146 | 126,122 | 235,374 | 105,248 | 119,149 | 126,063 |
| Total length | 97,732,475 | 97,693,426 | 100,316,629 | 100,092,240 | 98,482,702 | 98,276,062 | 100,589,888 | 98,998,995 | 98,257,134 | 98,768,164 |
| Total length (> 0 bases) | 105,342,642 | 105,303,593 | 105,198,781 | 105,157,542 | 105,270,006 | 105,189,432 | 111,859,095 | 111,116,108 | 106,856,977 | 101,249,996 |
| Total length (≥ 1 kb) | 88,117,765 | 88,100,505 | 96,449,445 | 95,482,963 | 92,087,443 | 91,536,870 | 96,547,165 | 93,790,943 | 94,640,693 | 95,182,359 |
| Total length (≥ 5 kb) | 62,424,381 | 62,421,352 | 83,553,563 | 79,011,990 | 72,887,505 | 71,909,125 | 77,686,457 | 70,717,763 | 74,807,576 | 74,886,448 |
| Total length (≥ 10 kb) | 42,323,674 | 42,322,500 | 69,063,332 | 62,882,217 | 54,920,296 | 53,924,860 | 59,770,766 | 50,856,038 | 57,435,528 | 55,876,172 |
| Total length (≥ 25 kb) | 14,422,596 | 14,422,263 | 38,717,465 | 32,953,824 | 23,823,003 | 23,302,388 | 28,963,671 | 19,101,307 | 28,042,606 | 23,994,724 |
| Total length (≥ 50 kb) | 2,369,995 | 2,369,995 | 15,817,193 | 12,717,169 | 5,731,300 | 5,620,109 | 8,617,684 | 4,293,011 | 8,968,358 | 5,749,503 |
| N50 | 8123 | 8129 | 18,437 | 15,302 | 12,067 | 11,744 | 13,415 | 10,430 | 13,234 | 12,294 |
| N75 | 3099 | 3103 | 7726 | 6125 | 4801 | 4633 | 5524 | 4309 | 5301 | 5193 |
| L50 | 2974 | 2972 | 1431 | 1669 | 2121 | 2163 | 1910 | 2467 | 1880 | 2104 |
| L75 | 7868 | 7860 | 3526 | 4262 | 5369 | 5507 | 4845 | 6193 | 4854 | 5247 |
| GC (%) | 35.39 | 35.39 | 35.35 | 35.35 | 35.38 | 35.38 | 35.54 | 35.56 | 35.83 | 35.83 |
