# Supplementary material for: GRASShopPER—An algorithm for de novo assembly based on GPU alignments
Source: PLoS One. 2018 Aug 16;13(8):e0202355. doi: 10.1371/journal.pone.0202355 (PMC6095601; doi:10.1371/journal.pone.0202355)
Supplement: S6 Table — (DOCX) [file pone.0202355.s006.docx]

**Table S6. Scaffolding of the data set *Homo sapiens* chromosome 14 for the assemblers GRASShopPER, SOAPdenovo2 and SGA with the combination of scaffolders SSPACE and SOAPdenovo2 (metrics calculated by QUAST)**

| Assembler | GRASShopPER | GRASShopPER | GRASShopPER | GRASShopPER | GRASShopPER | GRASShopPER | SGA | SGA | SOAPdenovo2 | SOAPdenovo2 |
| --- | --- | --- | --- | --- | --- | --- | --- | --- | --- | --- |
| Scaffolder | - | - | SSPACE | SSPACE | SOAPdenovo2 | SOAPdenovo2 | SSPACE | SOAPdenovo2 | SSPACE | SOAPdenovo2 |
| Postprocessing | no | yes | no | yes | no | yes |  |  |  |  |
| Genome fraction (%) | 92.277 | 92.275 | 91.922 | 92.275 | 91.955 | 91.92 | 88.586 | 88.277 | 89.033 | 89.582 |
| Duplication ratio | 1.038 | 1.038 | 1.043 | 1.038 | 1.04 | 1.038 | 1.013 | 1.007 | 1.007 | 1.006 |
| Largest alignment | 38,022 | 38,022 | 30,208 | 38,022 | 30,478 | 39,821 | 35,224 | 34,488 | 28,332 | 33,186 |
| Total aligned length | 86,648,379 | 86,645,202 | 86,674,089 | 86,645,202 | 86,477,849 | 86,174,358 | 81,213,834 | 80,463,428 | 81,184,217 | 81,612,904 |
| NG50 | 2500 | 2500 | 2466 | 2500 | 2414 | 3035 | 3040 | 3267 | 2432 | 2894 |
| NG75 | 1020 | 1020 | 1168 | 1020 | 1139 | 1311 | 1289 | 1332 | 1004 | 1206 |
| NA50 | 2665 | 2665 | 2530 | 2665 | 2491 | 3175 | 3480 | 3785 | 2804 | 3293 |
| NA75 | 1196 | 1197 | 1257 | 1197 | 1242 | 1458 | 1780 | 1909 | 1400 | 1623 |
| NGA50 | 2500 | 2500 | 2422 | 2500 | 2377 | 3000 | 3032 | 3256 | 2427 | 2890 |
| NGA75 | 1014 | 1013 | 1138 | 1013 | 1114 | 1286 | 1278 | 1323 | 1001 | 1200 |
| LG50 | 9417 | 9418 | 9897 | 9418 | 10,069 | 7899 | 8062 | 7466 | 9810 | 8271 |
| LG75 | 23,358 | 23,360 | 23,213 | 23,360 | 23,725 | 19,223 | 19,325 | 18,168 | 24,144 | 20,294 |
| LA50 | 8723 | 8722 | 9365 | 8722 | 9460 | 7411 | 6663 | 6067 | 8037 | 6903 |
| LA75 | 20,918 | 20,917 | 21,568 | 20,917 | 21,793 | 17,563 | 14,845 | 13,555 | 18,299 | 15,780 |
| LGA50 | 9435 | 9436 | 10,039 | 9436 | 10,212 | 7958 | 8080 | 7490 | 9819 | 8279 |
| LGA75 | 23,419 | 23,421 | 23,660 | 23,421 | 24,126 | 19,410 | 19,397 | 18,240 | 24,175 | 20,333 |
| Misassemblies |  |  |  |  |  |  |  |  |  |  |
| # misassemblies | 125 | 125 | 628 | 125 | 614 | 335 | 127 | 87 | 17 | 31 |
| # relocations | 119 | 119 | 620 | 119 | 609 | 328 | 125 | 86 | 16 | 28 |
| # translocations | 0 | 0 | 0 | 0 | 0 | 0 | 0 | 0 | 0 | 0 |
| # inversions | 6 | 6 | 8 | 6 | 5 | 7 | 2 | 1 | 1 | 3 |
| # misassembled contigs | 123 | 123 | 618 | 123 | 607 | 329 | 114 | 87 | 17 | 31 |
| Misassembled contigs length | 206,606 | 206,512 | 1,807,360 | 206,512 | 1,878,293 | 890,224 | 268,020 | 370,660 | 92,022 | 129,952 |
| # local misassemblies | 247 | 246 | 4204 | 246 | 345 | 271 | 3622 | 13 | 8 | 8 |
| # unaligned mis. contigs | 0 | 0 | 10 | 0 | 4 | 16 | 9 | 0 | 0 | 0 |
| Unaligned |  |  |  |  |  |  |  |  |  |  |
| # fully unaligned contigs | 292 | 291 | 334 | 291 | 317 | 972 | 108 | 108 | 120 | 818 |
| Fully unaligned length | 114,667 | 113,939 | 265,016 | 113,939 | 209,738 | 624,796 | 82,829 | 87,247 | 63,579 | 444,773 |
| # partially unaligned contigs | 10 | 10 | 105 | 10 | 65 | 181 | 29 | 13 | 3 | 6 |
| Partially unaligned length | 9791 | 9791 | 118,418 | 9791 | 61,981 | 153,848 | 27,409 | 9760 | 1655 | 3524 |
| Mismatches |  |  |  |  |  |  |  |  |  |  |
| # mismatches | 119,141 | 119,124 | 114,401 | 119,134 | 115,158 | 121,591 | 67,634 | 66,484 | 66,392 | 67,171 |
| # indels | 15,965 | 15,944 | 17,905 | 15,945 | 19,499 | 28,139 | 7998 | 9582 | 7385 | 17,946 |
| Indels length | 44,922 | 44,806 | 193,007 | 44,807 | 77,637 | 150,366 | 115,246 | 51,922 | 21,937 | 119,721 |
| # mismatches per 100 kb | 142.56 | 142.54 | 137.41 | 142.55 | 138.27 | 146.05 | 84.3 | 83.15 | 82.33 | 82.79 |
| # indels per 100 kb | 19.1 | 19.08 | 21.51 | 19.08 | 23.41 | 33.8 | 9.97 | 11.98 | 9.16 | 22.12 |
| # indels (≤ 5 bases) | 14,587 | 14,571 | 14,335 | 14,572 | 15,712 | 17,926 | 5824 | 6413 | 6552 | 9924 |
| # indels (> 5 bases) | 1378 | 1373 | 3570 | 1373 | 3787 | 10,213 | 2174 | 3169 | 833 | 8022 |
| # N's | 0 | 0 | 6271 | 0 | 52,711 | 228,289 | 5084 | 66,968 | 1 | 74,290 |
| # N's per 100 kb | 0 | 0 | 7.19 | 0 | 60.65 | 261.83 | 6.25 | 83.1 | 0 | 90.51 |
| Statistics without reference |  |  |  |  |  |  |  |  |  |  |
| no. contigs (> 0 bases) | 81,314 | 81,314 | 65,697 | 81,314 | 68,018 | 67,214 | 92,106 | 93,462 | 238,843 | 83,260 |
| no. contigs (≥250 bases) | 64,638 | 64,638 | 56,241 | 64,638 | 57,063 | 52,807 | 39,444 | 37,137 | 48,852 | 43,300 |
| no. contigs (≥ 1 kb) | 24,310 | 24,310 | 26,430 | 24,310 | 26,400 | 23,659 | 22,850 | 21,699 | 24,215 | 23,167 |
| no. contigs (≥ 5 kb) | 2988 | 2988 | 2805 | 2988 | 2744 | 3524 | 3601 | 3845 | 2845 | 3439 |
| no. contigs (≥ 10 kb) | 468 | 468 | 408 | 468 | 395 | 677 | 587 | 719 | 395 | 621 |
| no. contigs (≥ 25 kb) | 7 | 7 | 8 | 7 | 10 | 14 | 13 | 17 | 1 | 6 |
| no. contigs (≥ 50 kb) | 0 | 0 | 0 | 0 | 0 | 0 | 0 | 0 | 0 | 0 |
| Largest contig | 38050 | 38050 | 30444 | 38050 | 30478 | 39821 | 35224 | 34488 | 28332 | 33186 |
| Total length | 86,896,666 | 86,892,666 | 87,234,117 | 86,892,666 | 86,905,355 | 87,190,469 | 81,369,620 | 80,583,561 | 81,263,695 | 82,082,699 |
| Total length (> 0 bases) | 89,502,184 | 89,498,184 | 88,947,344 | 89,498,184 | 88,901,335 | 89,372,246 | 87,422,016 | 87,338,226 | 96,444,083 | 87,687,472 |
| Total length (≥ 1 kb) | 68,882,304 | 68,880,373 | 71,411,219 | 68,880,373 | 70,785,179 | 73,022,742 | 71,943,079 | 72,010,467 | 67,999,030 | 71,088,396 |
| Total length (≥ 5 kb) | 22,908,025 | 22,907,699 | 21,173,374 | 22,907,699 | 20,716,516 | 28,371,909 | 28,007,579 | 30,724,207 | 21,519,303 | 27,132,934 |
| Total length (≥ 10 kb) | 5,994,088 | 5,994,011 | 5,345,614 | 5,994,011 | 5,187,815 | 9,022,160 | 7,772,288 | 9,620,174 | 5,105,166 | 8,230,588 |
| Total length (≥ 25 kb) | 214,072 | 214,072 | 229,295 | 214,072 | 282,550 | 425,279 | 361,480 | 483,694 | 28,332 | 176,884 |
| Total length (≥ 50 kb) | 0 | 0 | 0 | 0 | 0 | 0 | 0 | 0 | 0 | 0 |
| N50 | 2673 | 2672 | 2577 | 2672 | 2536 | 3200 | 3494 | 3798 | 2807 | 3296 |
| N75 | 1200 | 1200 | 1292 | 1200 | 1269 | 1483 | 1788 | 1918 | 1403 | 1631 |
| L50 | 8706 | 8706 | 9235 | 8706 | 9328 | 7358 | 6650 | 6049 | 8030 | 6896 |
| L75 | 20,868 | 20,867 | 21,177 | 20,867 | 21,439 | 17,408 | 14,801 | 13,507 | 18,279 | 15,758 |
| GC (%) | 40.36 | 40.36 | 40.40 | 40.36 | 40.41 | 40.38 | 40.15 | 40.16 | 40.26 | 40.32 |
