# Supplementary material for: GRASShopPER—An algorithm for de novo assembly based on GPU alignments
Source: PLoS One. 2018 Aug 16;13(8):e0202355. doi: 10.1371/journal.pone.0202355 (PMC6095601; doi:10.1371/journal.pone.0202355)
Supplement: S1 Text — The list of GRASShopPER parameters. Description of how to run GRASShopPER. Parameters of all the methods used throughout computational experiment. (DOCX) [file pone.0202355.s007.docx]

GRASShopPER – an algorithm for *de novo* assembly based on GPU alignments – Supplementary material

Aleksandra Swiercz^1,2,3^, Wojciech Frohmberg^1,3^, Michal Kierzynka^1,3,4^, Pawel Wojciechowski^1,2,3^, Piotr Zurkowski^1,3^, Jan Badura^1,3^, Artur Laskowski^1,3^, Marta Kasprzak^1,2,3^, Jacek Blazewicz^1,2,3^

^1^ Institute of Computing Science, Poznań University of Technology, Poland

^2^ Institute of Bioorganic Chemistry, Polish Academy of Sciences, Poznań, Poland

^3^ European Centre for Bioinformatics and Genomics, Poznań, Poland

^4^ Poznań Supercomputing and Networking Center, Poland

1. **Algorithm parameters**

To run GRASShopPER it is strictly required to have installed and configured NVIDIA graphics card supporting compute capability 2.0 or higher. To assemble relatively short genomes it is also recommended to equip system with at least 32-64GB of RAM and 64GB of free disk space. In case of larger genomes we recommend the configuration with at least 512GB RAM and 1TB free disk space.

To run GRASShopPER, the assembler needs to be installed:

> cd <GRASSHOPPER-PATH>

> ./grasshopper compile

> sudo ./grasshopper install

Once the files are compiled, you may run GRASShopPER. A typical way to run the assembler is:

> grasshopper preprocess <pe-reads1.fastq> <pe-reads2.fastq> [opts...]

> grasshopper build <dataset> [opts...]

> grasshopper traverse <dataset> [opts...]

> grasshopper correct <dataset> [opts...]

> grasshopper trim <dataset>

> grasshopper scaffold <dataset> [opts...]

Each step needs information about the files that are being processed (*paired-end reads in fastq format*) or about the *dataset*, which is created in the first step of preprocessing the input files. The program is run with the default parameters which can be changed optionally. The optional parameters for every step are listed below.

preprocess

-ds=<dataset> – dataset name/path (default: common part of the file name containing paired-end reads)

-sg=<similar-genome> – (optional) genome file in fasta format to filter reads

-trimpath=<path-to-trimmomatic> – alternative to TRIMMOMATIC_PATH environment variable
-trimparams=<trimmomatic-params> – to alter the default Trimmomatic parameters

build

-ws=<window-size> – sets window size (default: 600)

-sc=<score-cutoff> – sets score cutoff (default: 50)

-e=<allowed-errors> – sets tolerance on errors between two reads (default: 0)

-kmer=<k-mer-size> – sets size of *k*-mer to compute characteristics (default: 6)

-pc=<characteristics-count> – count of partial characteristics (default: 3)

-ps=<characteristics-size> – the size of a single partial characteristic (default: 50)

-awa=<TRUE/FALSE> – enables enhancement that drastically increases search set of promising pairs to be verified (may be time consuming!) (default: FALSE)

-sli=<size> – the size of a shortest lexicographical index sequence (default: 20)

traverse

-fs=<forks-sensitivity> – sets the sensitivity of the forks detector (default: 6)

correct

-minconf=<value> – sets tolerance on distant paired-end reads (default: contigs_depth/7)

-maxrefs=<value> – sets the maximum number of distant paired end reads that after being exceeded we consider to add a new cut spot (default: contigs_depth/7)

scaffold

-m=<sspace/soap2> – chooses scaffolding tool (default: SSPACE)

-is=<insert-size> – sets expected insert size of the original paired-end reads file (if not given, it is calculated on the basis of mapping reads to contigs)

1. **GRASShopPER parameters for different data set**

*Candidatus* Microthrix parvicella

grasshopper preprocess ${YOUR_PATH}/SRR576810_1.fastq ${YOUR_PATH}/SRR576810_2.fastq –ds=Cmicro (-sg=<genome>)

grasshopper build Cmicro -sli=13 -ws=500 -awa=TRUE -e=4

grasshopper traverse Cmicro

grasshopper correct Cmicro -minconf=100 -maxrefs=0

grasshopper trim Cmicro

grasshopper scaffold Cmicro -m=sspace -is=312

*Caenorhabditis elegans*

grasshopper preprocess ${YOUR_PATH}/300_000_1.fastq ${YOUR_PATH}/300_000_2.fastq -ds=Cel (-sg=<genome>)

grasshopper build Cel

grasshopper traverse Cel -fs=2

grasshopper correct Cel -minconf=7 -maxrefs=7

grasshopper trim Cel

grasshopper scaffold Cel -m=soap2 -is=232

*Homo sapiens* chromosome 14

grasshopper preprocess ${YOUR_PATH}/frag_1.fastq ${YOUR_PATH}/frag_2.fastq –ds=chrom14

-trimparams="ILLUMINACLIP:${adapters/TruSeq3-PE.fa:2:30:10 SLIDINGWINDOW:30:20 LEADING:15 TRAILING:15 MINLEN:85" (-sg=<genome>)

grasshopper build chrom14

grasshopper traverse chrom14 -fs=100

grasshopper correct chrom14 -minconf=1 -maxrefs=1

grasshopper trim chrom14

grasshopper scaffold chrom14 -m=sspace -is=158

1. **Parameters for other methods used in the experiment**
   1. **CELERA** **(WGS 8.1)**.

Running Celera assembler requires a few steps:

- Creating *.frg files.

fastqToCA -insertsize 312 36 -libraryname Cmicro -technology illumina -mates $A,$B > Cmicro.frg

fastqToCA -insertsize 159 18 -libraryname Chr14 -technology illumina -mates $A,$B > Chr14.frg

fastqToCA -insertsize 232 56 -libraryname Cel -technology illumina -mates $A,$B > Cel.frg

where A and B are paths to fastq files.

- Constructing configure file “specfile”. All parameters are the same for all libraries except for the last parameter – file name

mbtThreads=16
ovlStoreMemory=70000
ovlThreads=16
ovlConcurrency=16
merOverlapperThreads=16
batThreads=16
cgwMergeFilterLevel=2
cnsConcurrency=16
merQCmemory=70000
ovlHashBits=27
ovlHashBlockLength=30000000
ovlRefBlockSize=7630000
frgCorrBatchSize = 1000000
frgCorrThreads   = 16
cleanup=aggressive
/path/to/file/*.frg

- Running the algorithm

runCA

- 1. **PLATANUS (v1.2.1)**

*Candidatus* Microthrix parvicella

platanus assemble  -o $results -f $IN1 $IN2 -t 4

platanus scaffold  -o $results -c `echo $results`_contig.fa -b `echo $results`_contigBubble.fa -a1 312 -d1 36 -n1 0 -IP1 $IN1 $IN2 -t 4

platanus gap_close -o $results -c `echo $results`_scaffold.fa -IP1 $IN1 $IN2 -t 4

*Caenorhabditis elegans*

platanus assemble  -o $results -f $IN1 $IN2 -t 4

platanus scaffold  -o $results -c `echo $results`_contig.fa -b `echo $results`_contigBubble.fa -a1 232 -d1 56 -n1 0 -IP1 $IN1 $IN2 -t 4

platanus gap_close -o $results -c `echo $results`_scaffold.fa -IP1 $IN1 $IN2 -t 4

*Homo sapiens* chromosome 14

platanus assemble  -o $results -f $IN1 $IN2 -t 4

platanus scaffold  -o $results -c `echo $results`_contig.fa -b `echo $results`_contigBubble.fa -a1 159 -d1 18 -n1 0 -IP1 $IN1 $IN2 -t 4

platanus gap_close -o $results -c `echo $results`_scaffold.fa -IP1 $IN1 $IN2 -t 4

where IN1, IN2 are fastq files, and results is an arbitrary prefix.

- 1. **SGA (v0.10.13)**

Configure file:

SGA_BIN=sga

OL=75

CPU=16

D=4000000

CK=41

COV_FILTER=2

MOL=55

R=10

MIN_PAIRS=5

MIN_LENGTH=200

SCAFFOLD_TOLERANCE=1

MAX_GAP_DIFF=0

$SGA_BIN preprocess --phred64 --pe-mode 1 -o $TEMP $IN1 $IN2

$SGA_BIN index -a ropebwt -t $CPU --no-reverse $TEMP

$SGA_BIN correct -k $CK --discard --learn -t $CPU -o reads.ec.k$CK.fastq $TEMP

$SGA_BIN index -a ropebwt -t $CPU reads.ec.k$CK.fastq

$SGA_BIN filter -x $COV_FILTER -t $CPU --homopolymer-check --low-complexity-check reads.ec.k$CK.fastq

$SGA_BIN fm-merge -m $MOL -t $CPU -o merged.k$CK.fa reads.ec.k$CK.filter.pass.fa

$SGA_BIN index -d 1000000 -t $CPU merged.k$CK.fa

$SGA_BIN rmdup -t $CPU merged.k$CK.fa

$SGA_BIN overlap -m $MOL -t $CPU merged.k$CK.rmdup.fa

$SGA_BIN assemble -m $OL -g $MAX_GAP_DIFF -r $R -o assemble.m$OL merged.k$CK.rmdup.asqg.gz

CTGS=assemble.m$OL-contigs.fa

GRAPH=assemble.m$OL-graph.asqg.gz

Running SGA:

sga-align --name celegans.pe $CTGS $IN1 $IN2

sga-bam2de.pl -n $MIN_PAIRS --prefix libPE celegans.pe.bam

sga-astat.py -m $MIN_LENGTH celegans.pe.refsort.bam > libPE.astat

$SGA_BIN scaffold -m $MIN_LENGTH --pe libPE.de -a libPE.astat -o scaffolds.n$MIN_PAIRS.scaf $CTGS

$SGA_BIN scaffold2fasta -m $MIN_LENGTH -a $GRAPH -o scaffolds.n$MIN_PAIRS.fa -d $SCAFFOLD_TOLERANCE --use-overlap --write-unplaced scaffolds.n$MIN_PAIRS.scaf

IN1, IN2 are input fastq files. TEMP is the temporary fastq file. The above configure file and running code was used to test *C. elegans* data set. The two other data sets except for one parameter in the configure file:

$SGA_BIN preprocess --phred64 --pe-mode 1 -o $TEMP $IN1 $IN2

The other data sets were run without parameter –phred64

Names of the temporary and output files were changed accordingly.

- 1. **SOAPdenovo2 (v2.04)**

Configure file:

max_rd_len=100

[LIB]

avg_ins=223

reverse_seq=0

asm_flags=3

rd_len_cutoff=100

rank=1

pair_num_cutoff=3

map_len=32

q1=/path/to/file/*.fastq

q2=/path/to/file/*.fastq

The only difference in the configure file is avg_ins:

*Candidatus* Microthrix parvicella : avg_ins=312

*Caenorhabditis elegans*: avg_ins=223

*Homo sapiens* chromosome 14: avg_ins=159

Running SOAPdenovo:

SOAPdenovo-63mer pregraph -s $config -K 51 -R -o $prefix -p 4

SOAPdenovo-63mer contig -g $prefix -R -p 4

SOAPdenovo-63mer map -s $config -g $prefix -p 4

SOAPdenovo-63mer scaff -g $prefix -F -p 4

Where prefix is an arbitrary prefix name for the results and config is the path to the configure file

- 1. **VELVET (v1.2.10)**

*Candidatus* Microthrix parvicella

velveth $hashDir 31 -shortPaired -fastq -separate $IN1 $IN2

velvetg $hashDir -ins_length 312 -ins_length_sd 36 -exp_cov 118 -scaffolding no  -min_contig_lgth 1000

*Caenorhabditis elegans*

velveth $hashDir 31 -shortPaired -fastq -separate $IN1 $IN2

velvetg $hashDir -ins_length 232 -ins_length_sd 56 -exp_cov 66 -scaffolding no  -min_contig_lgth 1000

*Homo sapiens* chromosome 14*:*

velveth $hashDir 31 -shortPaired -fastq -separate $IN1 $IN2

velvetg $hashDir -ins_length 159 -ins_length_sd 18 -exp_cov 26 -scaffolding no  -min_contig_lgth 1000

where IN1 and IN2 are the input files and hashDir is the name of the output directory

- 1. **SPAdes (3.11.1)**

*All datasets*:

./spades.py -k 21,33,55,77 --careful --pe1-1 IN1 --pe1-2 IN2 -o OUT

where IN1, IN2 are input fastq files and OUT is the output directory.
